# Spermine in semen of male sea lamprey acts as a sex pheromone

**DOI:** 10.1371/journal.pbio.3000332

**Published:** 2019-07-09

**Authors:** Anne M. Scott, Zhe Zhang, Liang Jia, Ke Li, Qinghua Zhang, Thomas Dexheimer, Edmund Ellsworth, Jianfeng Ren, Yu-Wen Chung-Davidson, Yao Zu, Richard R. Neubig, Weiming Li

**Affiliations:** 1 Department of Fisheries and Wildlife, Michigan State University, East Lansing, Michigan, United States of America; 2 College of Fisheries and Life Sciences, Shanghai Ocean University, Shanghai, China; 3 Department of Pharmacology and Toxicology, Michigan State University, East Lansing, Michigan, United States of America; Cornell University, UNITED STATES

## Abstract

Semen is fundamental for sexual reproduction. The non-sperm part of ejaculated semen, or seminal plasma, facilitates the delivery of sperm to the eggs. The seminal plasma of some species with internal fertilization contains anti-aphrodisiac molecules that deter promiscuity in post-copulatory females, conferring fitness benefits to the ejaculating male. By contrast, in some taxa with external fertilization such as fish, exposure to semen promotes spawning behaviors. However, no specific compounds in semen have been identified as aphrodisiac pheromones. We sought to identify a pheromone from the milt (fish semen) of sea lamprey (*Petromyzon marinus*), a jawless fish that spawns in lek-like aggregations in which each spermiating male defends a nest, and ovulatory females move from nest to nest to mate. We postulated that milt compounds signal to ovulatory females the presence of spawning spermiating males. We determined that spermine, an odorous polyamine initially identified from human semen, is indeed a milt pheromone. At concentrations as low as 10^−14^ molar, spermine stimulated the lamprey olfactory system and attracted ovulatory females but did not attract males or pre-ovulatory females. We found spermine activated a trace amine-associated receptor (TAAR)-like receptor in the lamprey olfactory epithelium. A novel antagonist to that receptor nullified the attraction of ovulatory females to spermine. Our results elucidate a mechanism whereby a seminal plasma pheromone attracts ready-to-mate females and implicates a possible conservation of the olfactory detection of semen from jawless vertebrates to humans. Milt pheromones may also have management implications for sea lamprey populations.

## Abstract 2

Male sea lampreys release via their ejaculates a highly specific and stimulatory sex pheromone, spermine, that activates a trace amine-associated receptor in the olfactory epithelium and attracts ready-to-mate females.

## Introduction

Semen is fundamental for sexual reproduction. Ejaculated semen contains sperm cells that are essential to fertilize eggs. The ejaculate confers reproductive advantages to males because the seminal plasma, the fluid compartment of semen, contains a myriad of molecules that alter the sexual attractiveness [1], physiology [2, 3], and sexual behavior of post-copulatory females [4]. For some animals with internal fertilization, ejaculate contains anti-aphrodisiac molecules that deter or inhibit other males from courting with copulated females to reduce sperm competition [1, 5]. Seminal molecules may also cause the females to cease mating and start laying eggs without further mating [6]. Many of these seminal molecules have been shown to be female hormones, or mimics of hormones, that regulate the reproductive physiology and behaviors of females [6]. Males gain reproductive advantages by investing in seminal molecules that suppress subsequent mating in already mated females [7].

In addition to the well-known anti-aphrodisiac function of seminal plasma in some species with internal fertilization, seminal plasma appears to be an aphrodisiac in some species with external fertilization in which seminal compounds stimulate mating behaviors. The milt (fish semen) of Pacific herring (*Clupea pallasii*) induces a series of reproductive behaviors in adults with elevated levels of steroid hormones despite no physical interaction occurring between the sexes when spawning [8]. In addition, female bitterling (*Rhodeus ocellatus*) increase egg deposition when exposed to milt [9]. The milt of these fishes likely contains pheromones that trigger the documented spawning behaviors. Evidence for this type of seminal pheromone remains limited to these 2 species. However, no specific molecules from semen have been identified with such a function, hampering a molecular understanding of how seminal plasma mediates reproductive behaviors.

We hypothesize that male sea lamprey, a basal vertebrate that spawns with multiple mates in a lek-like system, release a milt-derived pheromone. During their spawning season, sexually mature male lampreys that express milt (spermiating males) congregate on riverine gravel patches. Each spermiating male builds a nest and releases a sex pheromone from its gills that attracts sexually mature female (ovulatory) sea lampreys [10, 11]. In the spawning lek, each male defends a nest, while females move from nest to nest to spawn intermittently for approximately 1 week before mature adults die [11, 12]. During that period, the gill-released sex pheromone alone is insufficient to attract and retain the females on the nest, but spawning pairs have been observed to remain together for an extended duration [11]. This indicates that additional factors contribute to the maintenance of spawning aggregations. Because males typically release milt frequently for a week, seminal molecules released along with sperm would be a reliable, localized signal for the presence of actively spawning males in the vicinity, likely within a mating aggregation.

In this study, we discovered that spermine in sea lamprey milt acts as a specific semen-derived sex pheromone that promotes mating behaviors. This polyamine, initially discovered as crystals in human semen by Antonie van Leeuwenhoek in 1678 [13], evokes olfactory responses in teleost fish [14, 15] and possibly humans [16]. We reasoned that emitted spermine reveals sperm availability and thus can serve as a signal that benefits males seeking multiple mating partners. Our data indicate that sea lamprey seminal plasma contains high levels of spermine that stimulates the lamprey olfactory system and attracts ovulatory females at subpicomolar concentrations. We found that spermine activates a specific trace amine-associated receptor (TAAR)-like receptor (TAAR348), whereas an antagonist to this receptor inhibits olfactory and female behavioral responses to spermine. We conclude that spermine is a male pheromone and postulate that TAAR348 plays a role in mediating those responses to spermine.

## Results

### Spermine was present in seminal plasma of sea lampreys

**Fig 1:**
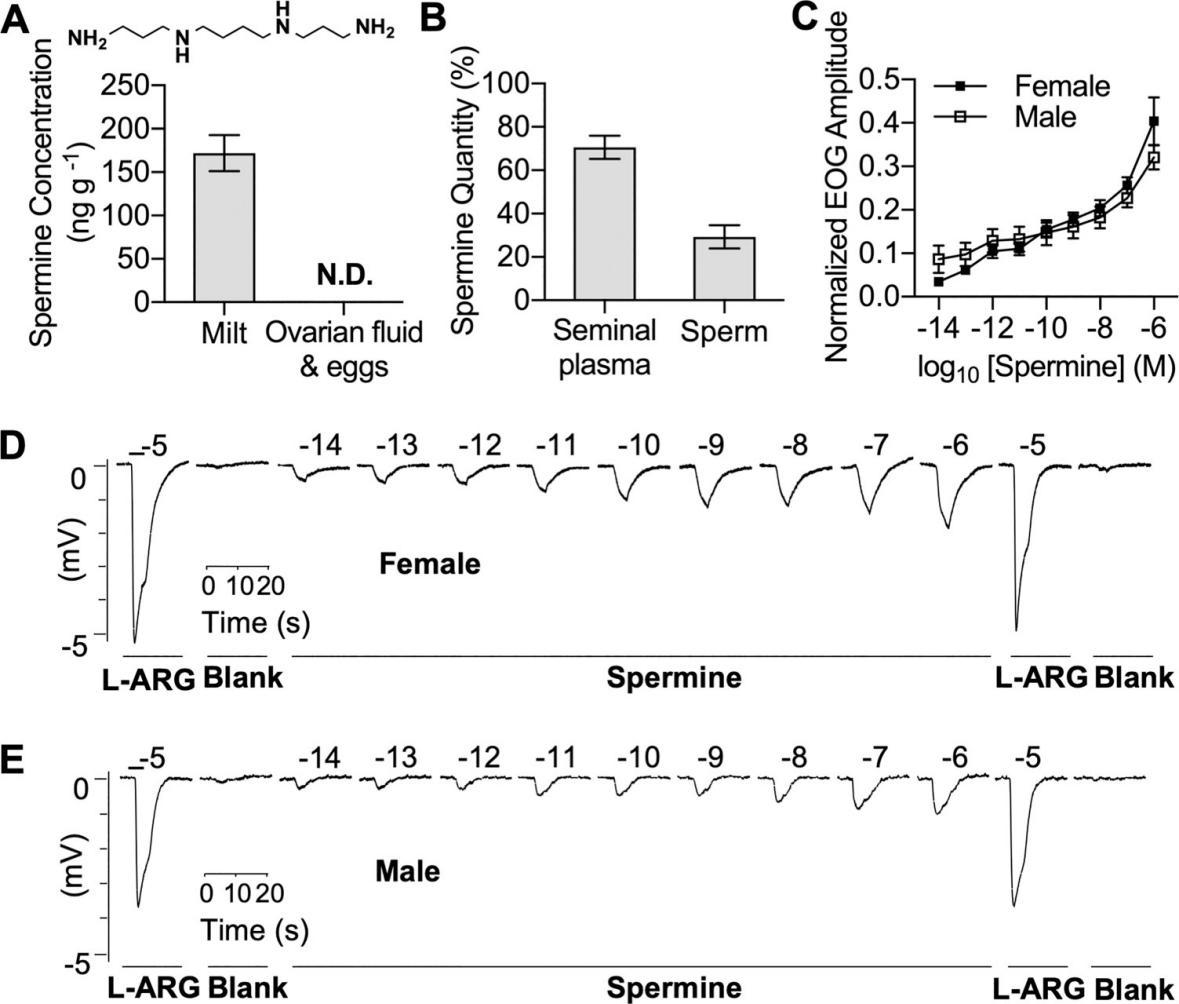
The seminal content and olfactory potency of spermine. (A) The concentration of spermine in sea lamprey milt and ovarian fluid with eggs (mean ± SEM, *n* = 6), measured with UHPLC–MS/MS. (Inset) Structure of spermine. (B) Percentage of spermine by weight in the milt components, seminal plasma and sperm, determined with UHPLC–MS/MS (mean ± SEM, *n* = 6). (C) Semi-logarithmic plot of normalized EOG amplitude (mean ± SEM) elicited by spermine (10^−15^ M to 10^−6^ M) in pre-ovulatory female (*n* = 10) and pre-spermiating male (*n* = 11) sea lampreys. The EOG response to spermine at each concentration was blank-subtracted and normalized to the response of 10^−5^ M _L_-ARG (standard) for each fish. (D) Representative EOG traces of pre-ovulatory female olfactory epithelia exposed to spermine at concentrations between 10^−15^ and 10^−6^ M. The number above each trace is the logarithmic value of the molar concentration of each stimulant. The bar above the _L_-ARG trace on the left represents the duration of odorant treatment. (E) EOG traces of pre-spermiating male olfactory epithelia exposed to spermine. Underlying data are available in S1 Data. blank, vehicle solution; EOG, electro-olfactogram; _L_-ARG, _L_-arginine; N.D., not detectable; UHPLC–MS/MS, ultrahigh performance liquid chromatography-tandem mass spectrometry.

Previous studies found that spermine (see structure in the inset of Fig 1A) is more abundant in semen than any other tissue or fluid [17, 18]. To measure emitted spermine from adult sea lampreys, we optimized an ultrahigh performance liquid chromatography-tandem mass spectrometry (UHPLC–MS/MS) assay and found that sea lamprey milt contained 172 ± 21 ng g^−1^ of spermine (mean ± SEM, *n* = 6; Fig 1A). When milt was fractionated into the 2 components—seminal plasma and sperm—around 70% of the spermine was detected in the seminal plasma (Fig 1B). In comparison, spermine was not detected in the mixture of expressed ovarian fluid and eggs from ovulatory females (Fig 1A). Spermine was also not detected in appreciable quantities in water conditioned with either ovulatory females or spermiating males, indicating it is not likely released from gills, as is known to be the case for other lamprey sex pheromones (S1 Table; limit of quantification of spermine from water samples: 0.02 ng mL^−1^) [11, 19]. Although spermine is a ubiquitous polyamine with wide-ranging cellular functions [13, 17, 18], our results indicate that milt—in particular the seminal plasma—is the main source of waterborne spermine from sexually mature male sea lampreys.

### Spermine potently stimulated the olfactory system and attracted ovulatory females

Spermine evoked concentration-dependent responses in adult sea lamprey olfactory epithelia (Fig 1C–1E), as recorded with electro-olfactogram (EOG). Spermine was highly stimulatory with a threshold of detection of 10^−14^ M for females (*p* = 0.005, paired *t* test, one tailed with a Bonferroni correction) and 10^−13^ M for males (*p* = 0.005, paired *t* test, one tailed with a Bonferroni correction; S1 Fig). The threshold of detection was also estimated by fitting a linear regression on log-transformed EOG data using the formula log(*N* + 1.5) = *a* log*C* + *b*, where *N* is the normalized response, *C* is the concentration, and *a* and *b* are constants following a previously described approach by Hubbard and colleagues [20]. The threshold of detection is the value for *x*, where *y* = 0.1761 (i.e., log 1.5; *N* = 0). Using this approach, we calculated the spermine threshold of detection to be less than 10^−14^ M for both females and males (females: 10^−14.9^ M and males: 10^−17.5^ M). We decided to report the more conservative results from the paired *t* test approach as the spermine thresholds of detection.

**Fig 2:**
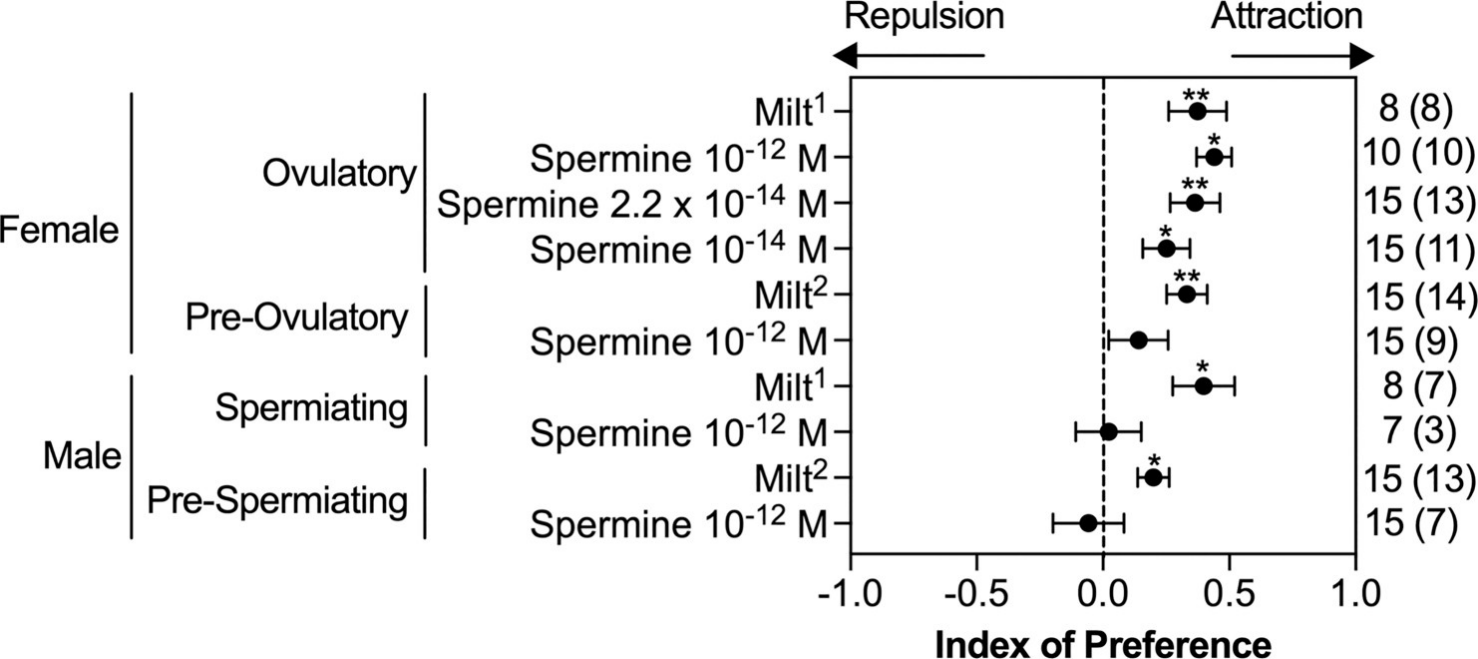
Adult sea lamprey behavioral responses to milt and spermine in a two-choice maze. The index of preference (mean ± SEM) was calculated as the value of [Ae ÷ (Ae + Be)–Ac ÷ (Ac + Bc)], where Bc is the cumulative amount of time spent in the control channel before odorant application, Be is the cumulative amount of time spent in the experimental channel before odorant application, Ac is the cumulative amount of time spent in the control channel after odorant application, and Ae is the cumulative amount of time spent in the experimental channel after odorant application in a two-choice maze (S2 Fig; see Materials and methods). A positive index value indicates attraction, and a negative index value indicates repulsion. Significance was evaluated using a Wilcoxon signed-rank test. *p < 0.05. **p < 0.01. The number in the parentheses indicates the number of test subjects spending more time in the experimental channel out of the total sample size. Milt was applied to produce a final spermine concentration of (1) 2.2 × 10^−14^ M or (2) 3.8 × 10^−14^ M in the maze. Underlying data are available in S1 Data.

To directly test the pheromone function of spermine, we examined the effect of spermine on adult sea lamprey behavior in a two-choice maze assay supplied with natural stream water (S2 Fig). Ovulatory females preferred the channel activated with milt when applied to produce a final spermine concentration in the maze of 2.2 × 10^−14^ M, compared with the channel with the vehicle (Fig 2, *p* = 0.008). Likewise, ovulatory females were attracted to spermine at 10^−14^, 2.2 × 10^−14^, and 10^−12^ M compared with the vehicle (Fig 2, *p* = 0.022, *p* = 0.003, *p* = 0.002, respectively). Ovulatory females had similar behavioral preferences for milt and spermine at 10^−14^, 2.2 × 10^−14^, and 10^−12^ M (*p* = 0.55).

The behavioral effect of spermine was maturation specific and sex specific. When applied at 10^−12^ M, spermine was not attractive over the vehicle for pre-ovulatory females, spermiating males, and pre-spermiating males (Fig 2, *p* = 0.359, *p* = 1.00, and *p* = 0.600, respectively). In contrast, milt itself attracted all adults, including pre-ovulatory females, spermiating males, and pre-spermiating males, over the vehicle (Fig 2, *p* < 0.001, *p* = 0.023, and *p* = 0.004, respectively). Our chemical, electrophysiological, and behavioral evidence demonstrates that spermine is a sex pheromone that specifically attracts ovulatory females.

### Spermine activated a single TAAR-like receptor expressed in HEK293T cells

**Fig 3:**
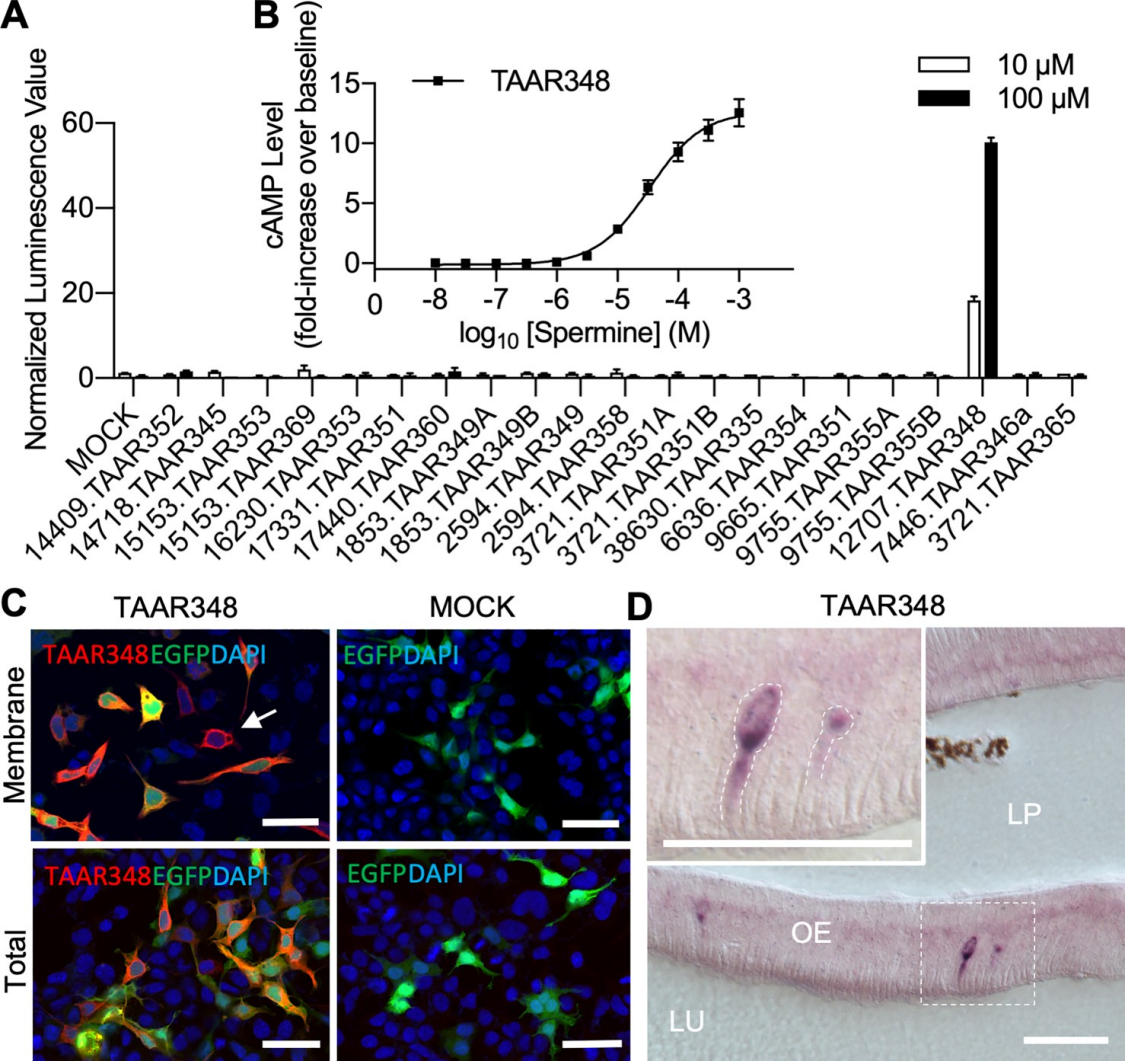
Spermine activated sea lamprey TAAR348. (A) HEK293T cells were incubated with TAAR plasmids or mock (empty vector) along with a CRE-luciferase reporter vector for 48 hours and subsequently stimulated with 10 or 100 μM spermine for 4 hours. Luciferase activity was indicated by luminescence value and was normalized to the response from the control stimuli DMSO (mean ± SEM, *n* = 2). (B) As an inset to (A), (B) shows spermine-induced cAMP production in HEK293T cells expressing TAAR348. The cAMP levels were measured with a TR-FRET assay and normalized to the cAMP level in buffer-treated cells (mean ± SEM, *n* = 5). Fold increase over basal was calculated as [(measured cAMP − basal cAMP) ÷ basal cAMP] − 1. (C) TAAR348 targeted to HEK293T cell membrane as shown by the immunostained Rho-tag antibody (red) for total (bottom panel, with permeation using Triton X-100) or membrane-bound (top panel, without permeation) expression. Receptors located on the cell surface appeared as red rings around the nucleus (denoted with arrow). The nucleus was counterstained with DAPI (blue). EGFP was used as the negative control to evaluate transfection efficiency (green). Scale bar: 50 μm. (D) Representative olfactory receptor neurons expressing *taar348* (purple), labeled with a DIG-labeled antisense RNA probe in a cross-sectional view of the main olfactory epithelium of an adult female sea lamprey. The section was counterstained with Nuclear Fast Red. Scale bar: 50 μm. Underlying data are available in S1 Data. cAMP, cyclic-adenosine monophosphate; CRE, cyclic-adenosine monophosphate response element; DIG, digoxigenin; EGFP, enhanced green fluorescent protein; HEK293T, human embryonic kidney 293T; LP, lamina propria; LU, lumen; OE, olfactory epithelium; TAAR, trace amine-associated receptor; TR-FRET, time-resolved fluorescence energy transfer.

Next, we sought to characterize the olfactory chemoreceptor(s) that detects spermine. We focused our search on TAARs, a family of receptors known to mediate olfactory responses to mono- and di-amines [21, 22] and to modify specific behaviors in vertebrates [21, 23]. The sea lamprey genome contains genes encoding 26 TAAR-like receptors [24]. We expressed these receptors in human embryonic kidney 293T (HEK293T) cells, a modified HEK293 cell line that expresses the simian virus 40 (SV40) large T antigen that efficiently amplifies the transcription vector with the receptor gene inserts, as described by Zhuang and colleagues [25] and measured the responses to spermine. This heterologous expression system has been used to express various G-protein-coupled receptors (GPCRs), including chemoreceptors from a variety of species. Of the 26 TAAR-like receptors, 21 appropriately targeted to the cell membrane, and of these, only TAAR-like 348 (TAAR348, hereafter) showed robust responses to 10 μM spermine (Fig 3A). Spermine did not activate any of the 22 sea lamprey odorant receptors (ORs) or 3 sea lamprey vomeronasal type 1 receptors (V1Rs) expressed in HEK293T cells (S3 Fig).

Spermine stimulated cyclic-adenosine monophosphate (cAMP) production in HEK293T cells expressing TAAR348 in a concentration-dependent manner, but not in mock-transfected cells or cells with other TAARs (Fig 3B and 3C). TAAR348 gave robust responses to spermine with a half-maximal response occurring at 34 ± 1 μM (mean ± SEM; half-maximal effective concentration [EC_50_]). TAAR348 also responded to 4 other amine variants (S4A Fig); however, they were at least 30-fold less potent and did not produce maximal responses at the highest concentration tested (30 μM; S4B Fig).

In situ hybridization results showed that *taar348* transcripts were located in a sparse population of olfactory sensory neurons (OSNs) in adult sea lampreys (Fig 3D and S5 Fig). The *taar348* probe hybridized to mRNA in a small subset of OSNs in the lamellae in middle sections along the rostral-caudal axis of the main olfactory epithelium but were not contained to certain olfactory epithelial zones as positive staining was sparsely distributed along the dorsoventral and lateral axes. Labeled intact neurons showed a tall morphology [26], homologous to teleost ciliated OSNs [27], with the cell bodies situated deeper in the olfactory epithelium and long dendrites projecting towards the epithelial surface. In comparison, no labeling was observed either in intermediate OSNs, homologous to teleost microvillous OSNs, which have a characteristic plump cell body and an intermediate soma position, or in short OSNs, homologous to teleost crypt OSNs, which have a rounded, egg-shape and are situated in the most apical layer of the epithelium [26, 27]. These data demonstrate that TAAR348 located in the olfactory epithelium is a cognate receptor of spermine with a high level of specificity.

### A specific antagonist of TAAR348 reduced olfactory and nullified behavioral responses to spermine

**Fig 4:**
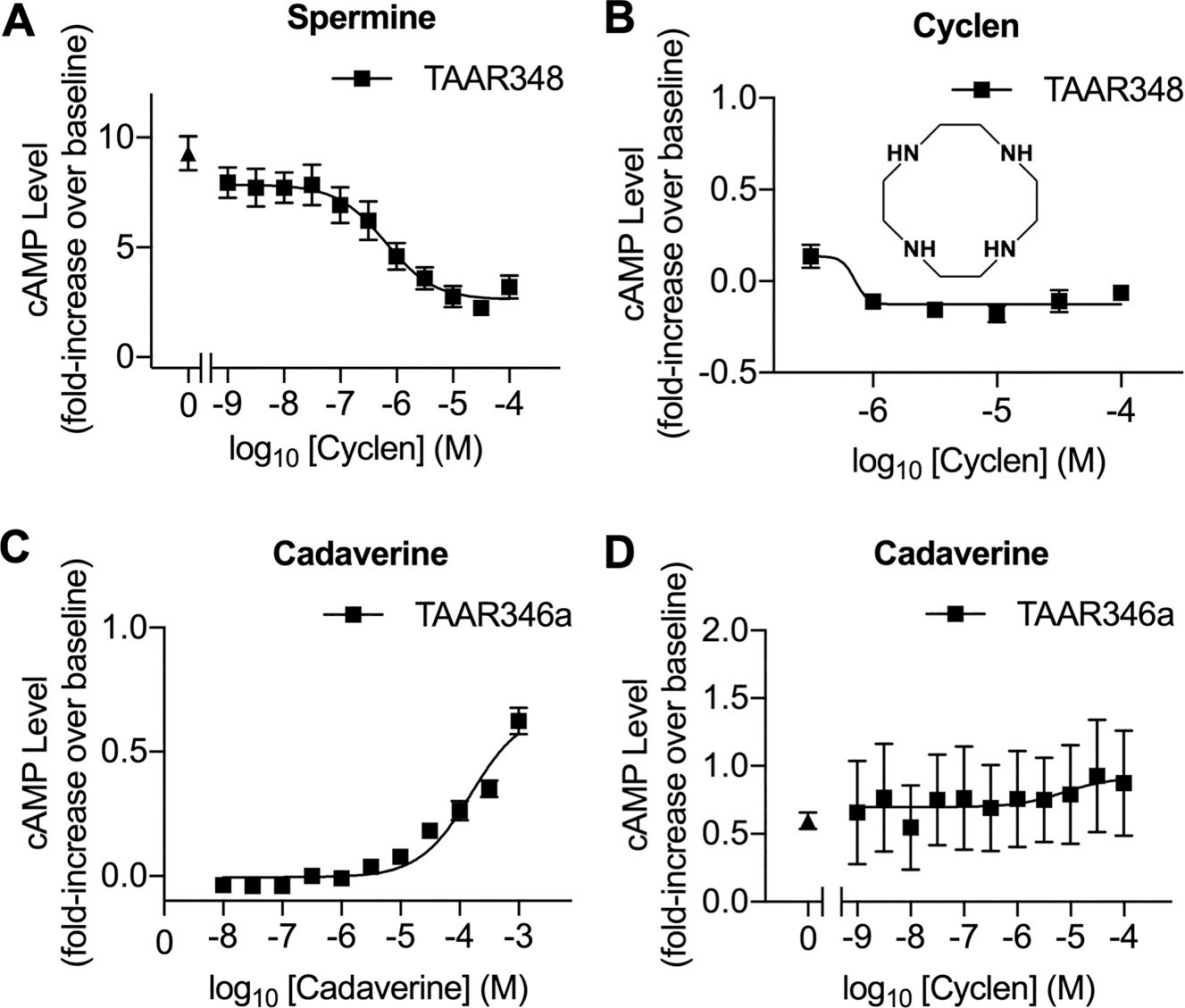
Cyclen antagonized spermine-induced cAMP responses to receptor TAAR348. (A) Cyclen treatment inhibited cAMP production induced by 0.1 mM spermine in TAAR348-expressing HEK293T cells as measured with a TR-FRET assay and normalized to the cAMP level in buffer-treated cells (mean ± SEM, *n* = 5). The filled triangle represents cAMP production induced by 0.1 mM spermine in the cells without exposure to cyclen. Fold increase over basal was calculated as [(measured cAMP − basal cAMP) ÷ basal cAMP] − 1. (B) Cyclen did not induce cAMP production in HEK293T with TAAR348 (mean ± SEM; reads from triplicate wells). (Inset) Structure of cyclen (1,4,7,10-tetraazacyclododecane). (C) Cadaverine induced cAMP production in a concentration-dependent manner in HEK293T cells expressing another sea lamprey TAAR-like gene—TAAR346a (mean ± SEM, *n* = 5). (D) Cyclen treatment did not inhibit cAMP production induced by 1 mM of cadaverine in HEK293T cells expressing TAAR346a. The filled triangle represents the cAMP production induced by 1 mM of cadaverine in the cells without exposure to cyclen (mean ± SEM, *n* = 5). Underlying data are available in S1 Data. cAMP, cyclic-adenosine monophosphate; HEK293T, human embryonic kidney 293T; TAAR, trace amine-associated receptor; TR-FRET, time-resolved fluorescence energy transfer.

Because genetic editing methods are not feasible in adult sea lamprey [28], we utilized pharmacologic tools to examine the role of TAAR348 in mediating the observed olfactory and behavioral responses to spermine in sea lampreys. We screened 22 structural analogs of spermine (S2 Table) as potential antagonists of the TAAR348-mediated cAMP responses to spermine (S6A Fig). We found that cyclen (1,4,7,10-tetraazacyclododecane; see structure in the inset of Fig 4B) blocked the spermine-induced cAMP-stimulating activity. It inhibited spermine-induced cAMP production in HEK293T cells with TAAR348 in a concentration-dependent manner with a half-maximal inhibitory concentration (IC_50_) in the nanomolar range, 0.6 ± 0.1 μM (Fig 4A). Cyclen appears to be a pure antagonist because it did not induce cAMP production when added alone (Fig 4B). To assess the specificity of cyclen as an antagonist of TAAR348, we tested cyclen on HEK293T cells expressing another sea lamprey TAAR-like receptor (TAAR346a), which we found responded to cadaverine (Fig 4C). TAAR346a and TAAR348 shared 47% identity at the amino acid level (S7 Fig); however, cyclen treatment did not inhibit cAMP production induced by cadaverine in TAAR346a-expressing HEK293T cells (Fig 4D). The incomplete maximal inhibition (approximately 70% in Fig 4A) is consistent with an action as a negative allosteric modulator that may contribute to the specificity of its actions. Our data indicate that cyclen is a potent and selective antagonist for TAAR348.

**Fig 5:**
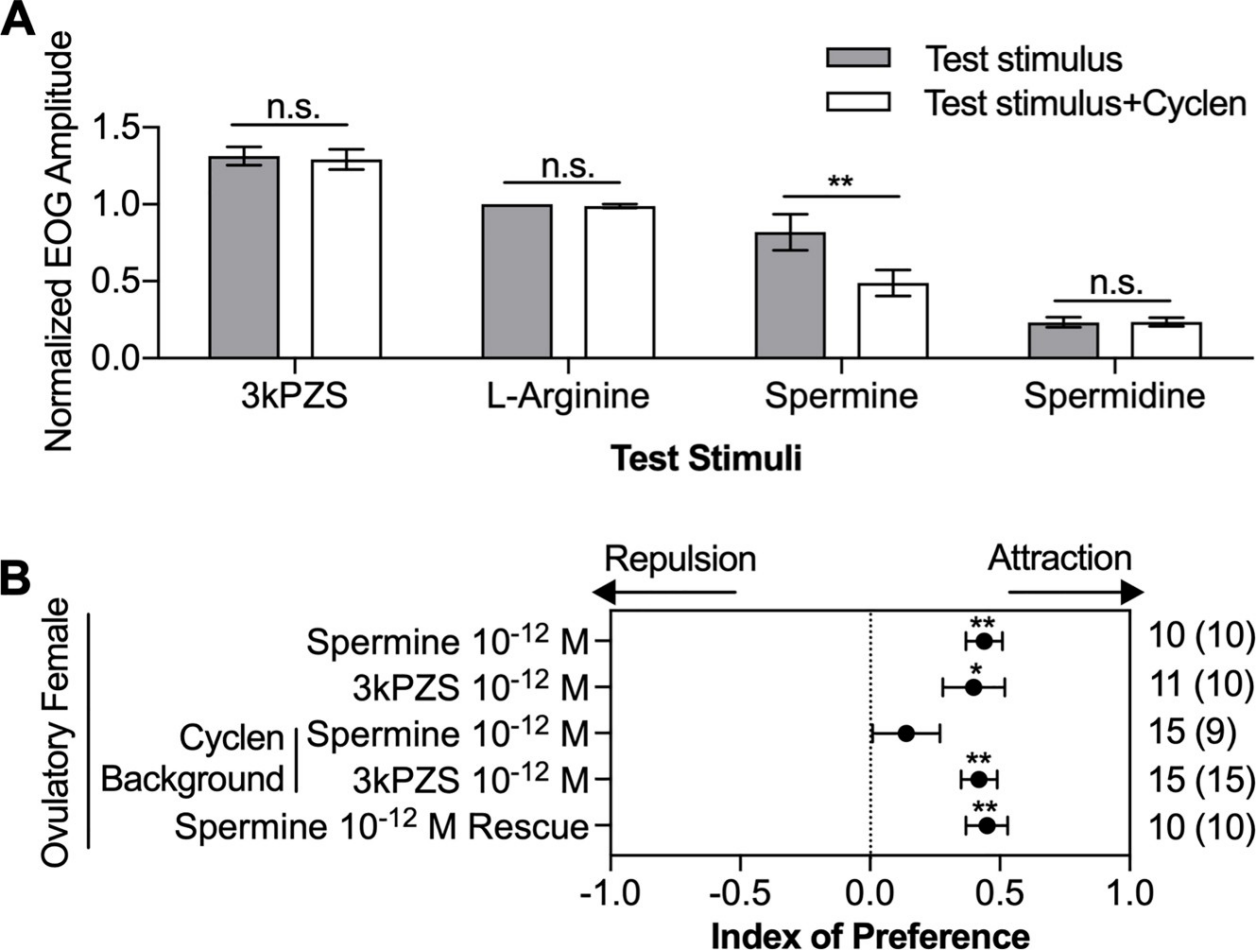
Cyclen impeded spermine-induced olfactory and behavioral responses. (A) Cyclen treatment (10^−5^ M) reduced the EOG response magnitude to spermine (10^−5^ M) but not to 3kPZS (10^−7^ M), spermidine (10^−5^ M), or _L_-arginine (10^−5^ M). EOG responses were blank-subtracted and normalized to the amplitude of the responses to 10^−5^ M _L_-arginine (mean ± SEM, *n* = 5). The difference in responses for each stimulus before (gray) and during (white) exposure of the naris to cyclen was evaluated with a paired *t* test. (B) Ovulatory female sea lamprey behavioral responses to spermine and 3kPZS (positive control) in the absence and presence of 10^−12^ M cyclen (cyclen background) in a two-choice maze assay (S2 Fig). The effect of cyclen was reversible because the attraction to spermine was rescued when cyclen application ceased. For the definition of the index of preference, see Materials and methods. A positive index value indicates attraction, and a negative index value indicates repulsion. Significance was evaluated using a Wilcoxon signed-rank test. The number in the parentheses indicates the number of test subjects spending more time in the experimental channel out of the total sample size. *p < 0.05, **p < 0.01. Underlying data are available in S1 Data. EOG, electro-olfactogram; n.s., not significant; 3kPZS, 3-keto petromyzonol sulfate.

Cyclen treatment also reduced olfactory responses to spermine in adult sea lampreys. The olfactory epithelium was exposed to cyclen for 5 minutes, and then the olfactory responses to cyclen mixed with various other stimuli—including spermine, _L_-arginine [29], 3-keto petromyzonol sulfate (3kPZS), which is a male sea lamprey sex pheromone released through the gills [19], and spermidine, which is the immediate precursor of spermine biosynthesis [13]—were recorded. Treating the olfactory epithelium with cyclen reduced the response to spermine (*p* = 0.005) but not to other odorants (*p* > 0.10; Fig 5A).

Likewise, cyclen nullified attraction of ovulatory females to spermine. When both maze channels were perfused with cyclen (10^−12^ M), spermine at 10^−12^ M did not induce a preference in ovulatory females over the vehicle (Fig 5B, *p* = 0.33). As expected, cyclen did not alter the behavioral response of ovulatory females to the gill-released pheromone 3kPZS. 3kPZS attracted ovulatory females in the absence or presence of cyclen (Fig 5B, *p* = 0.014 and *p* < 0.001, respectively). Furthermore, inhibition of the behavioral response to spermine by cyclen was reversible. When cyclen application ceased, ovulatory females were attracted to spermine (Fig 5B, *p* = 0.002). The modification of the electrophysiological and behavioral responses to spermine in lampreys exposed to cyclen is consistent with the cyclen-mediated inhibition of the cAMP responses to spermine in HEK293T cells expressing TAAR348, the only receptor of the 46 tested that responds to spermine.

### A spermine receptor agonist induced olfactory and behavioral responses comparable to those induced by spermine

**Fig 6:**
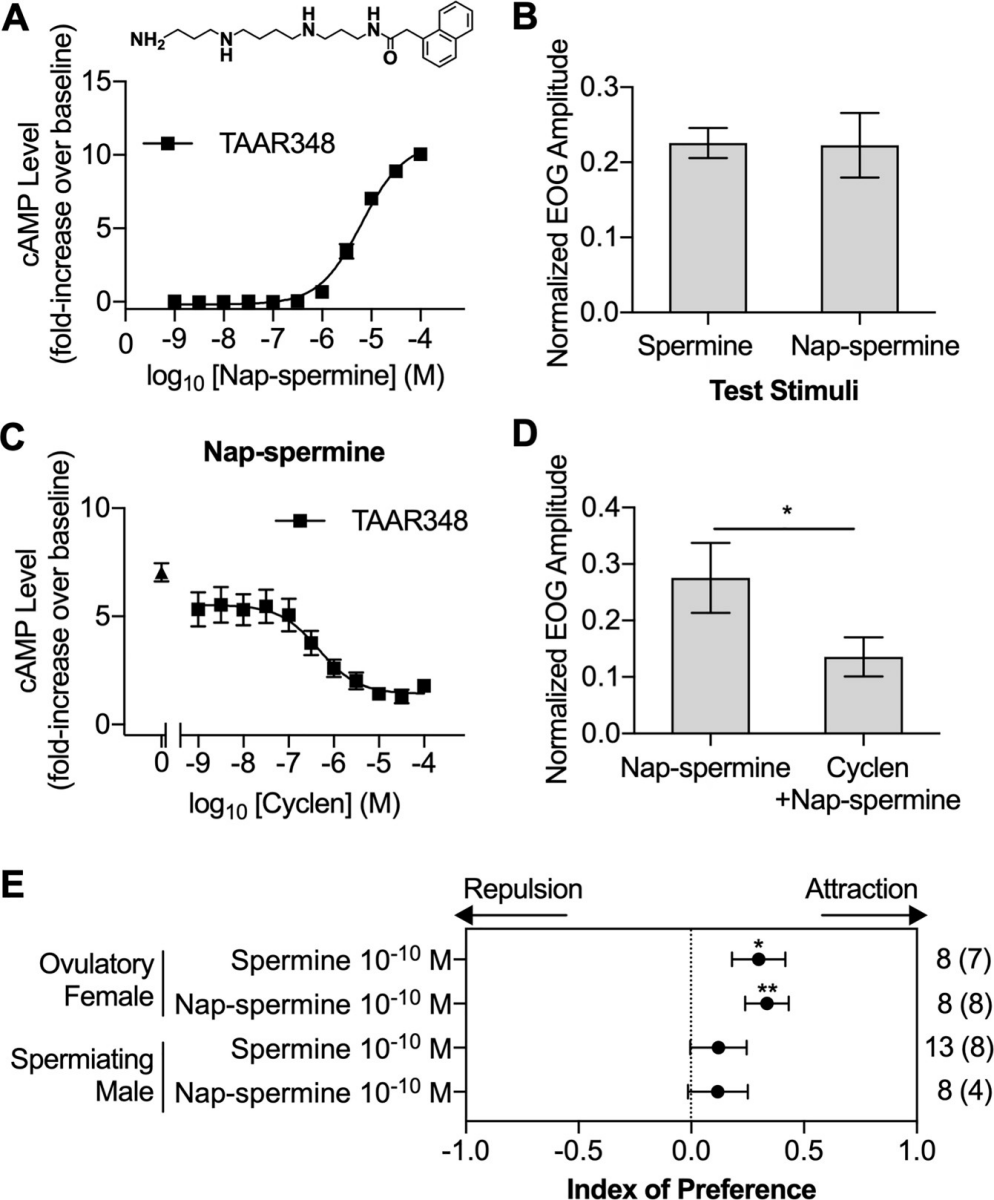
Nap-spermine, an agonist of spermine receptor TAAR348, induced responses virtually identical to those induced by spermine. (A) Nap-spermine induced cAMP production in a concentration-dependent manner in TAAR348-expressing HEK293T cells (mean ± SEM, *n* = 5). The cAMP assay was done as in Fig 3B. (Inset) Structure of nap-spermine. (B) EOG responses to 10^−7^ M spermine and 10^−7^ M nap-spermine (mean ± SEM, *n* = 12 and *n* = 6, respectively). Normalized EOG responses were blank-subtracted and normalized to the amplitude of the responses to 10^−5^ M _L_-arginine. (C) Cyclen inhibited cAMP production induced by 10^−5^ M nap-spermine in HEK293T cells with TAAR348. The filled triangle represents the cAMP production induced by TAAR348-expressing HEK293T cells exposed to only 10^−5^ M nap-spermine (mean ± SEM, *n* = 5). (D) Cyclen treatment (10^−5^ M) reduced the EOG response magnitude to nap-spermine (10^−6^ M). Cyclen exposure conditions are the same as in Fig 5A. EOG responses were blank-subtracted and normalized to the amplitude of the responses to 10^−5^ M _L_-arginine (mean ± SEM, *n* = 5). (E) Adult sea lamprey behavioral responses to spermine and nap-spermine in a two-choice maze (S2 Fig). For definition of the index of preference, see Materials and methods. A positive index value indicates attraction, and a negative index value indicates repulsion. Significance was evaluated using a Wilcoxon signed-rank test. The number in the parentheses indicates the number of test subjects spending more time in the experimental channel out of the total sample size. *p < 0.05, **p < 0.01. Underlying data are available in S1 Data. cAMP, cyclic-adenosine monophosphate; EOG, electro-olfactogram; HEK293T, human embryonic kidney 293T; nap-spermine, 1-naphthylacetyl spermine; TAAR, trace amine-associated receptor.

In our final step to characterize a possible role of TAAR348 in mediating responses to spermine, we reasoned that another TAAR348 agonist, if found, should replicate the effects of spermine at the receptor, olfactory epithelia, and behavioral levels. From 22 structural analogs tested (S2 Table), we found that 1-naphthylacetyl spermine (nap-spermine; see structure in the inset of Fig 6A) was an agonist of TAAR348 (S6B Fig). Nap-spermine induced robust cAMP production in cells expressing TAAR348, with an EC_50_ of 5 ± 1 μM, comparable to that of spermine (Fig 6A). As expected, nap-spermine induced EOG responses of magnitudes comparable to those induced by spermine (Fig 6B). Moreover, cyclen inhibited the nap-spermine–induced cAMP production with an IC_50_ of 0.5 ± 0.3 μM (Fig 6C), remarkably similar to the IC_50_ for spermine-induced activity. As was the case for spermine, prolonged exposure of the olfactory epithelium to cyclen reduced the olfactory response magnitude to nap-spermine (Fig 6D, *p* = 0.01). Consistent with the behavioral results of spermine, nap-spermine attracted ovulatory females when compared with the vehicle (*p* = 0.008) but did not attract spermiating males (*p* = 0.38; Fig 6E). In summary, cyclen consistently nullified the receptor, olfactory, and behavioral responses induced by spermine, whereas nap-spermine consistently induced responses that replicated those induced by spermine. These results, coupled with the lack of spermine-induced responses for 45 other sea lamprey olfactory chemoreceptors that we have tested, is consistent with a specific role for TAAR348 in mediating the pheromone function of spermine.

## Discussion

In this study, we show that sea lamprey milt is a source of the chemical cue spermine, which attracts ovulatory females, representing a chemically defined, semen-derived pheromone that stimulates reproductive behaviors. Pheromones are described as chemical signals that elicit stereotyped responses in certain conspecifics [6, 30]. Our chemical analyses linked the source of waterborne spermine to milt. Our biological assays demonstrated remarkable olfactory potency of spermine and a strong behavioral preference of ovulatory females for spermine. In addition, we showed that a sea lamprey TAAR-like receptor, TAAR348, responds to spermine when expressed in a heterologous system. Furthermore, a newly identified, selective TAAR348 antagonist reduced the olfactory response and completely nullified the female behavioral response to spermine. A distinct TAAR348 agonist replicated spermine effects, producing virtually identical responses at the receptor, olfactory epithelia, and whole-organism behavioral levels. These lines of evidence demonstrate that spermine is a pheromone that originates in the seminal plasma and attracts ready-to-mate female sea lampreys. Our data also support a model in which TAAR348 plays a key role in mediating the pheromone function of spermine.

The dichotomy in behavioral responses of adult female and male sea lampreys to spermine is consistent with our hypothesis that ovulatory females use spermine to identify males actively releasing sperm in a lek-like spawning aggregation. Spermine as a male signal is highly specific in its effects on individuals of the same species, attracting only ovulatory females and no other adults. On a typical nest, a male and one to several females often stay together and spawn many times [11]. Therefore, a signal such as spermine, released through the act of spawning, may contribute to the maintenance of the temporary spawning groups and attract additional female mates. Although spermine elicits distinct sex specific behavioral responses, both male and female sea lampreys detect spermine at the olfactory level with comparable potencies. This phenomenon is consistent with those responses observed for sex pheromones in sea lamprey [19, 31], goldfish [32, 33], tilapia [34, 35], and some insects [36].

In contrast to the highly specific pheromone effect of spermine on ovulatory females, milt itself attracted both males and females of various maturation stages. Although this has not been documented previously in other species, such a broad effect of milt on behaviors of conspecific adults is consistent with the promiscuous spawning system of sea lamprey. Other males and females, mature or immature, may detect milt odors to spy on spawning habitat and potential mates engaging in spawning activities. Therefore, we speculate that these additional odorants from semen may be chemical cues that do not directly benefit the releasing male. In fact, a previous study has shown that immature sea lampreys en route to spawning grounds track odorants of spermiating males [37]. Males may benefit from their attraction to milt because an aggregate of males in a lek-like system will collectively release more pheromone and attract more females than any one male can alone. The milt odorant(s) that attracts males and pre-ovulatory females remains to be identified. Future studies—particularly in natural spawning streams—on these aspects of milt function may further explain the fitness benefit of the incredible chemical complexity known to exist in seminal plasma [2, 7].

The remarkable olfactory potency of spermine is consistent with the effects of other sea lamprey pheromones and is likely a critical factor that enables this compound to function as a pheromone. Sea lampreys spawn in rapids with water velocities up to 1.5 m s^−1^ [11], where emitted milt containing spermine is likely quickly diluted, forming a steep spermine concentration gradient immediately downstream of a nest. Adult sea lampreys appear to be well adapted to detect and respond to spermine at a wide range of concentrations. Sea lamprey have the ability to smell spermine from 10^−14^ or 10^−13^ M to 10^−6^ M, whereas the ovulatory females showed preference to spermine from 10^−14^ to 10^−10^ M. The behavioral attraction to a wide range of concentrations is consistent with those demonstrated for the lamprey mating pheromone [10]. The olfactory detection threshold of spermine is 1 to 2 orders of magnitude lower than those of either the lamprey mating or migratory pheromone [19, 38, 39]. Spermine is detected at much lower concentrations in vivo (i.e., EOG and behavioral responses) compared with in vitro (i.e., TAAR348 expressed in HEK293T), suggesting more optimized conditions for receptor expression and function in native sea lamprey neurons or possibly the presence of additional types of spermine receptors. Notably, spermine evokes olfactory responses in goldfish and zebrafish, 2 species that spawn in more stagnant water, but only at much higher concentrations of 10^−8^ M and 10^−6^ M, respectively [14, 15]. Whether spermine functions as an attractive pheromone in these 2 species has not been examined. Although spermine is known to be an odorant, its influence on behavior has not been examined in other animals.

The function of spermine as an odorant may be widespread because it is likely present in semen of many vertebrates. Pheromones are not necessarily species specific, and pheromone functions can be maintained through many mechanisms of species isolation [6]. If spermine is found to function as a pheromone in other species, it might exemplify convergent evolution of pheromone structures in distant species [40] constrained by common biochemical processes [6]. It is not surprising that spermine has been co-opted as a sex pheromone, which often evolve from compounds once originally intended for other functions linked to mate choice [41]. Spermine is abundant in semen [13, 17] and critical to male fertility [42]. It is possible that spermine is also critical to male fertility and sperm mobility in sea lamprey, as has been shown in mammals [16, 43]. An ovulatory female lamprey should be keenly attuned to such a signal that is a reliable indicator for sperm availability to maximize the fertilization rate and her fitness [44].

Our molecular and cellular data indicate that TAAR348 appears to be a narrowly tuned olfactory receptor. TAAR348 showed robust, concentration-dependent responses only to spermine and not to other biogenic amines. Spermine as a polyamine represents a new type of ligand for TAAR receptors, which have been shown to detect mono- and di-amines previously [45, 46]. Notably, the gene family encoding TAAR-like receptors first appeared in lampreys and evolved independently from the TAAR gene family of jawed vertebrates [47]. It would be interesting to determine whether spermine activates TAAR receptors of teleost fishes and humans [14, 15, 21, 22, 47].

Inhibition of multiple spermine functions by cyclen treatment shows that TAAR348 likely plays a role in sea lamprey responses to spermine. Cyclen specifically and potently inhibited TAAR348-mediated cAMP production in response to spermine but did not inhibit another polyamine-responsive receptor (TAAR346a). In the olfactory epithelia, cyclen treatment reduced the EOG responses to spermine but not to other odorants, including a known male lamprey pheromone 3kPZS and spermidine, a precursor to spermine. More importantly, cyclen treatment blocked the behavioral responses of ovulatory females to spermine, but not to 3kPZS, at equal concentrations (10^−12^ M). Furthermore, the spermine response was restored when cyclen treatment was removed. In addition, another TAAR348 agonist, nap-spermine, replicated the effects of spermine by inducing virtually identical responses at the receptor, olfactory epithelia, and behavioral levels. All this evidence lends strong support for the model that TAAR348 mediates olfactory detection of spermine. Our data, however, cannot exclude the possible involvement of additional receptors in the detection of spermine, because the cyclen treatment reduced but did not completely eliminate EOG responses to spermine. Odorant perceptions are often encoded by the combinatorial activation of multiple receptors, particularly with TAARs [48]. In the future, the role of TAAR348 should be further examined through gene knockout experiments, when such techniques become feasible in adult lampreys [28].

Based on chemical, physiological, and behavioral evidence, we conclude that spermine is a male pheromone in sea lamprey. This discovery implicates a new strategy that male animals use to recruit mates through the release of chemical cues in ejaculates, as opposed to the commonly recognized strategy for males to deny further courtship to copulated females through seminal plasma compounds. Notably, the sea lamprey is an abundant and destructive invasive species in the Laurentian Great Lakes [49, 50] despite being imperiled in many countries throughout its native range [49]. Spermine, and our identification of cyclen as a spermine receptor antagonist, may provide approaches for either control or conservation of sea lamprey populations.

## Materials and methods

### Ethics statement

All procedures involving sea lampreys were approved by the Michigan State University Institutional Animal Use and Care Committee (03/14-054-00 and 02/17-031-00). Sea lamprey used for EOG recordings were anesthetized with exposure to 3-aminobenzoic acid ethyl ester (MS222; 100 mg/L; Sigma-Aldrich, St. Louis, MO) and immobilized with an intramuscular injection of gallamine triethiodide (3 mg/kg of body weight, in 0.9% saline). Gills were continuously irrigated with aerated water containing MS222 for the duration of recording, and sea lampreys were euthanized with MS222 followed by decapitation.

### Animals

Reagents in the Materials and methods were purchased from Sigma-Aldrich (St. Louis, MO) unless stated otherwise. Immature adult sea lampreys (pre-spermiating males and pre-ovulatory females) were captured in tributaries of the Laurentian Great Lakes by the United States Fish and Wildlife Service and Fisheries and Oceans Canada; were transported to the US Geological Survey Hammond Bay Biological Station, Millersburg, Michigan; and were held in 500 to 1,000 L aerated flow-through tanks maintained at 15 to 19°C. Pre-spermiating male (232.1 g ± 29.3, 487.8 mm ± 14.2; mean ± SEM) and pre-ovulatory female (266.3 g ± 21.2, 509.6 mm ± 13.4) sea lampreys used for EOG recordings were transported to the University Research Containment Facility at Michigan State University, East Lansing, Michigan, held in flow-through tanks (250 L) supplied with aerated, chilled well water maintained at 7 to 9°C, and were used in April and May 2016 to 2018. To produce sexually mature adults (spermiating male and ovulatory females) for spermine quantification and behavioral assays conducted in June and July 2016 to 2018, immature adults were held in cages constructed of polyurethane mesh and plastic pipe (0.5 m^3^) located in the lower Ocqueoc River, Millersburg, Michigan, to allow natural maturation. These animals were monitored daily for signs of sexual maturation [11].

### Cell line

HEK293T cells were maintained at 37°C with 5% CO_2_ and grown in Dulbecco’s Modified Eagle Medium (DMEM; Hyclone; Logan, UT) supplemented with 10% fetal bovine serum (FBS; Gibco; Waltham, MA) without antibiotic.

### Quantification of spermine

Ovulatory female and spermiating male sea lampreys were separated by sex and held for 14 hours in two 500-L tanks supplied with continuous flow-through, aerated Lake Huron water at 17.7°C before samples were collected for the quantification of spermine. Each lamprey was transferred to a 20-L bucket supplied with continuous flow-through, aerated Lake Huron water and acclimated for 1 hour. All water was drained from the bucket, and 3 L of deionized water with an air stone was added. After 1 hour, the lamprey was removed from the bucket, and a conditioned water sample was collected. The water samples were concentrated 50 times using a CentriVap Cold Trap with CentriVap Concentrator (Labconco; Kansas City, MO) and then stored at −20°C until further analysis. Milt (sperm with seminal plasma) and eggs with ovarian fluid were collected from spermiating males or ovulatory females, respectively, by applying gentle pressure to the abdomen, resulting in expression of gametes from the cloacal opening. A subset of the milt samples were centrifuged (10 minutes, 1,020*g* at 4°C) to separate the seminal plasma from the sperm. The seminal plasma (supernatant) was transferred to a new tube. The samples were stored at −80°C until further analysis.

To prepare the stock solutions of spermine (≥99%, 85590) and [^2^H_8_] -spermine (Sp-*d*_8_, 95%, 705330), each compound was dissolved in water/methanol (7:3, v/v) at a concentration of 1 mg mL^−1^. The stock solution of internal standard (IS hereafter; Sp-*d*_8_) was further diluted with water/methanol (7:3, v/v) to 500 ng mL^−1^; 10-μl IS was added to each sample before extraction as described by Magnes and colleagues [51] with modification. Briefly, each 1 mL sample spiked with IS was treated with trichloroacetic acid (TCA; 4%, 100 μL) [52], vortexed (5 minutes), and centrifuged (10 minutes, 10,000*g*), resulting in 500 μL supernatant that was mixed with 500 μL deionized water. Subsequently, sodium carbonate buffer (0.1 M [pH 9], 125 μL) and isobutyl chloroformate (25 μL) were added and incubated at 35°C for 15 minutes. The isobutyl chloroformate residue was cleaned [53] and reconstituted to 100 μL with the initial mobile phase.

A Waters ACQUITY H-Class UHPLC system connected to a Waters Xevo TQ-S triple quadrupole mass spectrometer was used to detect spermine in the conditioned water and gamete samples (Waters Corp.; Milford, MA). The mobile phase consisted of water (containing 0.1% formic acid) as (A) and acetonitrile (containing 0.1% formic acid) as (B). A Waters BEH C18 column (2.1 × 50 mm, 1.7 μm particle size) coupled with an Acquity UHPLC column in-line filter kit (0.2 μm filter) was used. Samples were separated with a gradient program at a flow rate of 250 μL min^−1^ for 12 minutes at 35°C: 70% A for 1 minute, decreased to 0% A from 1 to 7 minutes, and then maintained at 0% A from 7.01 to 9.0 minutes, increased to 70% A from 9.0 to 9.01 minutes, and then maintained at 70% A to 12 minutes for column equilibrium. The sample injection volume was 10 μL. Spermine was detected by Multiple Reaction Monitoring (MRM) mode and processed using Masslynx 4.1 software (Waters Corp.; http://www.waters.com/waters/en_US/MassLynx-MS-Software/nav.htm?locale=en_US&cid=513662). The UHPLC–MS/MS parameters were optimized for the transition of the spermine analyte as follows: [M + H]^+^ *m/z* 603.4, MRM *m/z* 603.4 > 154.9, cone voltage 49 V, collision energy 40 eV, and retention time 6.92 minutes; and for the spermine-*d*_*8*_ analyte as follows: [M + H]^+^ *m/z* 611.4, MRM *m/z* 611.4 > 163.1, cone voltage 36 V, collision energy 40 eV, and retention time 6.92 minutes.

The UHPLC effluent was introduced into the mass spectrometer with electrospray ionization in the negative mode. The electrospray ionization mass spectrometry (ESI–MS/MS) parameters were set as follows: capillary voltage, 2.60 kV; extractor voltage, 5 V; source temperature, 150°C; desolvation temperature, 500°C; and desolvation gas flow, 800 L h^−1^ (N_2_, 99.9% purity). Argon (99.9999% purity) was introduced as the collision gas into the collision cell at a flow rate of 0.15 mL min^−1^. Data were collected in centroid mode with a scan range of 50 to 1,000 *m/z*. The dwell time established for each transition was 0.2 seconds, and the interscan delay was set at 20 ms. Data acquisition was carried out using Masslynx 4.1 software and processed using TargetLynx (Waters Corp.). The limit of spermine quantification with the UHPLC–MS/MS was 1.0 ng mL^−1^. Because the water samples were concentrated 50 times, the limit of spermine quantification of the water samples was 0.02 ng mL^−1^.

### EOG recordings

EOG setup and recordings were conducted following established procedures by Li and colleagues [54] to determine whether the adult sea lamprey olfactory organ was sensitive to spermine. Sea lampreys were anesthetized with MS222 (100 mg L^−1^), immobilized with an intramuscular injection of gallamine triethiodide (3 mg kg^−1^ of body weight, in 0.9% saline), and placed in a V-shaped plastic stand. Gills were continuously irrigated with aerated water containing 50 mg L^−1^ MS222. The olfactory lamellae were surgically exposed by removing the skin on the surface of the olfactory capsule. The differential EOG response to each test stimulus was recorded using borosilicate electrodes filled with 0.04% agar in 0.9% saline connected to solid state electrodes with Ag/AgCl pellets (model ESP-M15N; Warner Instruments; Hamden, CT) in 3M KCl. The recording electrode was placed between 2 olfactory lamellae and adjusted to maximize the response to _L_-arginine standard while minimizing the response to the blank control (vehicle in charcoal-filtered water handled in the same way as stimulus solution but without the addition of spermine), and the reference electrode was placed on the external skin near the naris. Electrical signals were amplified by a NeuroLog system (model NL102; Digitimer, England, UK), filtered with a low-pass 60 Hz filter (model NL125; Digitimer), digitized by Digidata 1440A (Molecular Devices, San Jose, CA), and recorded on a PC running AxoScope 10.4 software (Molecular Devices; http://mdc.custhelp.com/app/answers/detail/a_id/18779/~/axon%E2%84%A2pclamp%E2%84%A2-10-electrophysiology-data-acquisition-%26-analysis-software-download).

For the concentration-response recordings, the olfactory epithelia of sea lampreys were exposed to 10^−15^ M to 10^−6^ M solutions of spermine. A 10^−3^ M stock solution of spermine in water/methanol (1:1, v:v) was prepared, stored at −20°C, and then serially diluted with filtered water to yield 10^−15^ M to 10^−6^ M solutions. A 10^−2^ M stock solution of _L_-arginine in deionized water was prepared, stored at 4°C, and diluted with filtered water to yield a 10^−5^ M solution. A 10^−5^ M _L_-arginine solution was introduced to the olfactory epithelium for 4 seconds, and the response was recorded to correct for variations in olfactory sensitivity among fish. The olfactory epithelium was flushed with filtered water for 2 minutes, and then the blank control was introduced and recorded. Next, the test stimulus starting at 10^−15^ M to 10^−6^ M was applied in log_10_ molar increments, recorded, and flushed. Blank control and 10^−5^ M _L_-arginine standard were measured repeatedly (approximately after every 3 concentrations of stimuli) throughout each recording session. The EOG response magnitudes were measured in mV. The normalized EOG response was calculated as normalized EOG amplitude = (Rt − Rb) ÷ (Ra − Rb), where Rt is the response magnitude to the test stimulus, Rb is the response magnitude to the blank, and Ra is the response magnitude to 10^−5^ M _L_-arginine. The responses to 10^−5^ M _L_-arginine standard (mean ± SEM, male: 2.75 ± 0.08, female: 3.41 ± 0.08) and blank (male: 0.17 ± 0.01, female: 0.13 ± 0.01) were comparable to previous studies [54–56]. The threshold of detection was defined as the lowest concentration in which the test stimulus elicited a larger response than the blank (paired *t* test, one tailed with a Bonferroni correction for 4 comparisons, α = 0.0125).

For the cyclen treatment to the olfactory epithelium experiments, the EOG responses to the test stimuli (spermine, _L_-arginine, 3kPZS [Bridge Organics Co., Vicksburg, MI]), spermidine, and nap-spermine) were first recorded in a similar manner as the concentration-response recordings. Then, the naris was continuously exposed to 10^−5^ M cyclen for 5 minutes. Next, the EOG responses to mixtures of each test stimulus with the adapting solution of 10^−5^ M cyclen were recorded. The naris was rinsed with charcoal-filtered water for 2 minutes, and then the responses to the test stimuli were recorded to ensure recovery of the olfactory system. The normalized EOG response of each test stimuli before and during cyclen treatment was evaluated with a two-tailed paired *t* test.

### Two-choice maze behavioral assay

The behavioral preferences of the sea lampreys to the test stimuli were evaluated using a two-choice maze assay that was described in a previous study [54] (S2 Fig). Briefly, a single lamprey was introduced to the acclimation cage at the downstream end of the maze for 5 minutes. The lamprey was released, and the cumulative amount of time the lamprey spent in each channel was recorded for 10 minutes (pretreatment period before odorant application). The test stimulus was introduced to a randomly chosen channel and vehicle (water when milt was test stimulus and methanol for other test stimuli) to the other channel at constant rates of 200 ± 5 mL min^−1^. The test stimulus and vehicle were pumped into the maze for 5 minutes. The cumulative amount of time the lamprey spent in each channel was recorded for 10 minutes while continuing to apply the test stimulus and vehicle (odorant application period). The maze was flushed with water for 10 minutes before the start of the next experiment to remove any remaining test stimulus. The time spent in the control (Bc) and experimental (Be) channel before odorant application and in the control (Ac) and experimental (Ae) channel after odorant application were used to calculate an index of preference for each trial as defined by index of preference = [Ae ÷ (Ae + Be)–Ac ÷ (Ac + Bc)] [54]. The index of preference was evaluated using a Wilcoxon signed-rank test (α = 0.05) to determine whether the index of preference was significantly different from zero. Differences were considered significant at *p* < 0.05. A significant positive value of the index of preference indicated attraction. A significant negative value of the index of preference indicated repulsion. A nonsignificant value of the index of preference indicated neutral. The trial was discarded if the sea lamprey failed to enter the control and experimental channel for at least 10 seconds during the 10-minute period before the odorant was applied because this was an indication of strong side bias or inactivity.

To assess the influence of cyclen on behavioral responses of ovulatory females to spermine, a modified behavioral assay was followed. Cyclen (10^−12^ M) was applied to the experimental and control channel during the acclimation and pretreatment period. The cumulative amount of time the lamprey spent in each channel during the pretreatment period was recorded. Then, a mixture of either cyclen (10^−12^ M) and spermine (10^−12^ M, test stimulus) or cyclen (10^−12^ M) and 3kPZS (10^−12^ M, control; Bridge Organics) was introduced to a randomly chosen channel and a mixture of cyclen (10^−12^ M) and vehicle to the other channel for 5 minutes. Then, the cumulative amount of time the lamprey spent in each channel was recorded for 10 minutes while the treatments continued to be administered. An index of preference was calculated as described above. To determine whether the influence of cyclen on the spermine behavioral response was reversible, cyclen (10^−12^ M) was applied to the experimental and control channel during the acclimation and pretreatment periods. Next, cyclen application stopped, and spermine application (10^−12^ M) started. Spermine was applied to the experimental channel and vehicle to the control channel for the treatment period. The cumulative amount of time the lamprey spent in each channel during the pretreatment and treatment period was recorded, and an index of preference was calculated.

### High-throughput ligand screening of TAARs

The open reading frames of *TAARs* were mined from the sea lamprey genome assembly (Pmarinus_7.0) [24]. A total of 26 *TAARs* were annotated, cloned from genomic DNA, and introduced into Rho-pCMV modified from pCMV-Tag-2B (211172; Agilent Technologies, Santa Clara, CA) by introducing a Rho-tag (the first 21 amino acids of bovine rhodopsin) at the N terminal to replace the intrinsic flag-tag [25].

HEK293T cells were seeded at 5,500 cells per well on a 384-well plate and cotransfected with 5-μL DNA-transfection mixture that contained 5 ng of a TAAR plasmid, 5 ng pCI-mRTPs (provided by Dr. H. Matsunami, Duke University, Durham, NC), and 5 ng pEGFP-N1 (6085–1, Clontech, Mountain View, CA). The empty plasmid, pCI-mRTPs, and pEGFP-N1 were cotransfected as a negative control. The plate was incubated for 48 hours at 37°C with 5% CO_2_, fixed with 3 μL 37% formaldehyde per cell for 15 minutes at room temperature (RT), washed with 50 μL PBS 3 times, and incubated with 25 μL blocking buffer (1× PBS) with 5% bovine serum albumin (BSA) for 1 hour at RT. Subsequently, 25 μL mouse monoclonal anti-rhodopsin antibody (1:500, MABN15, Millipore, Burlington, MA) was added to each well and incubated at 4°C overnight. The antibody solution was aspirated, washed with 50 μL PBS 3 times, and incubated with Alexa Fluor 594 goat anti-mouse IgG (1:500, A11005, Thermo Scientific, Waltham, MA) for 1 hour at RT. Nuclei were counterstained with DAPI (1:1,000, D1306, Thermo Scientific). Images were acquired at 200× magnification under Cytation 3 Cell Imaging Multi-Mode Reader (BioTek, Winooski, VT) with DAPI, green fluorescent protein (GFP), and Texas Red filters. Additional images of TAAR348 membrane and total expression patterns were sequentially acquired on a Nikon A1 laser scanning confocal microscope with DAPI, GFP, and Texas Red filters. Images were sequentially acquired in single XY-plane and merged. Results indicated that 21 TAARs targeted to the plasma membrane, which were used in subsequent screening experiments.

The initial high-throughput screening (HTS) was performed in 384-well plates as described by Zhuang and colleagues [25] with the following modifications. For the reverse transfection, 5 μL DNA-transfection mixture (20 ng CRE-Luciferase vector pGL4.29 [E8471; Promega, Madison, WI], 5 ng of a TAAR plasmid, 5 ng pCI-mRTPs, 5 ng pCI-G_αolf_ [provided by Dr. H. Matsunami]), and 5,500 HEK293T cells in 25 μL 0.5% FBS DMEM medium were added to each well. The plates were incubated for 48 hours at 37°C with 5% CO_2_. Each stimulus solution (either 150 nL of 2 mM in DMSO for a final concentration of 10 μM or 150 nL of 20 mM in DMSO for a final concentration of 100 μM) was dispensed into a designated well using Biomek FXP liquid handling automation workstation (Beckman Coulter; Brea, CA). The negative control stimulus was 150 nL DMSO. Plates were incubated for 4 hours at 37°C with 5% CO_2_. Luciferase activity was measured using Steady Glo Luciferase Assay System (E2520, Promega), and luminescence was read on a Synergy Neo multi-mode microplate reader (BioTek). Luciferase activity was normalized by DMSO-stimulated luminescence value with the following formula: (Luc induced by Ligand) ÷ (Luc induced by DMSO).

### Assay for cAMP production

The cAMP production assay was performed in 384-well plates as described in LANCE Ultra cAMP Kit manual (TRF0263, PerkinElmer; Waltham, MA) to characterize the cAMP production induced in HEK293T cells expressing TAAR348. Briefly, HEK293T cells were seeded in a 100-mm dish with 3 × 10^6^ cells in 10-mL complete culture medium (DMEM medium with 10% FBS and 1× Antibiotic-Antimycotic; Gibco) and incubated for 24 hours at 37°C with 5% CO_2_. Cells were then transfected with 5 μg pGL4.29, 1 μg pCI-mRTPs, 1 μg pCI- G_αolf_, and 1 μg TAAR plasmid and incubated at 37°C with 5% CO_2_ for 24 hours. Transfected cells were detached with Versene (15040066, Thermo Scientific) and transferred to 384-well plates at 5 μL (2,000 cells) per well; 5 μL of the 2× spermine serial dilutions were added to each well and incubated for 30 minutes at RT. Afterwards, 5 μL 4× Eu-cAMP tracer working solution and 5 μL 4× ULight-anti-cAMP working solution were added to each well and incubated for 1 hour at RT. Plates were read with the Synergy Neo multi-mode microplate reader for TR-FRET emissions at 620 nm (as internal reference) and 665 nm (as biological response). The ratio of 665/620 allows normalization for the well-to-well variability and interference due to assay components.

### Screening of agonists and antagonists of TAAR348

Twenty-two spermine structural analogs (S2 Table) were screened to identify another agonist of TAAR348 using a strategy similar to the initial HTS with the following modifications. Briefly, 3 × 10^6^ HEK293T cells were seeded in 10 mL complete culture medium and incubated for 24 hours on day 1. On day 2, the cells were cotransfected with 5 μg pGL4.29, 1 μg pCI-mRTPs, 1 μg pCI-G_αolf_, and 1 μg TAAR plasmid and then incubated for 24 hours. On day 3, the transfected cells were harvested and then reseeded in 384-well plates at a density of 9,000 cells per well in 30 μL 0.5% FBS DMEM medium and incubated for 24 hours. On day 4, 150 nL of each analog solution (2 mM in DMSO; S2 Table), or 150 nL DMSO as a negative control, was dispensed into a designated well and subsequently incubated for 4 hours. Luciferase activity was measured as the indicator for receptor activity as described in the HTS.

To identify antagonists of the spermine-induced responses in HEK293T cells expressing TAAR348, the screening procedure followed that of the agonist screening from day 1 through day 3. On day 4, 150 nL of analog solution (2 mM in DMSO, S2 Table), or 150 nL DMSO as a negative control, was dispensed into each well and incubated for 30 minutes at 37°C with 5% CO_2_. Afterward, 5 μL 6 × 10^−5^ M spermine was added to each well and incubated for 4 hours at 37°C with 5% CO_2_ before the luciferase activity as described in the HTS.

To examine the inhibition of cyclen on spermine-induced cAMP production in HEK293T cells expressing TAAR348, HEK293T cells were transfected with 5 μg pGL4.29, 1 μg pCI-mRTPs, 1 μg pCI- G_αolf_, and 1 μg TAAR348. TAAR348-expressing HEK293T cells were stimulated with successive additions of cyclen serial dilutions and 0.1 mM spermine. The cAMP production assay was performed as described for characterization of TAAR348. Subsequently, Eu-cAMP tracer and ULight-anti-cAMP were added, incubated for 1 hour at RT, and read for TR-FRET emissions at 620 nm (as internal reference) and 665 nm (as biological response). To examine the inhibition specificity of cyclen, the effect of cyclen on another sea lamprey TAAR (TAAR346a) induced cAMP production was assessed. The cAMP production assay was performed as described for the cyclen inhibition of spermine-induced responses with the following modifications: HEK293T cells were transfected with 5 μg pGL4.29, 1 μg pCI-mRTPs, 1 μg pCI- G_αolf_, and 1 μg TAAR346a. TAAR346a-expressing HEK293T cells were stimulated with successive additions of cyclen serial dilutions and 1 mM cadaverine (TAAR346a agonist; Sigma-Aldrich D22606). Subsequently, Eu-cAMP tracer and ULight-anti-cAMP were added, incubated, and read for TR-FRET emissions at 620 nm (as internal reference) and 665 nm (as biological response).

### In situ hybridizations of *taar348*

Probes (approximately 350–400 bp) were designed based on the coding region of *taar348*. The amplified DNA fragments were cloned into a pGEM-T vector (A3610, Promega), and the sequences were verified. Each plasmid was linearized using restriction enzyme Nco (antisense probe) or Spe (sense probe) and used for synthesis of digoxigenin (DIG)-labeled RNA probes with DIG RNA labeling kit (SP6/T7; 11175025910, Roche; Basel, Switzerland). In situ hybridization was conducted following previously described methods by Chung-Davidson and colleagues [57]. Briefly, 20-μm frozen sections of olfactory epithelium were hybridized with RNA probes (3 ng μL^−1^) overnight at 65°C in the hybridization solution (50% deionized formamide, 1× Denhart's solution, 5% dextran sulfate, 750 mM sodium chloride, 25 mM ethylenediaminetetraacetic acid, 25 mM piperazine-N, Nʹ-bis-2-ethanesulfonic acid, 0.25 mg mL^−1^ fish sperm DNA, 0.25 mg mL^−1^ poly A acid, and 0.2% sodium dodecyl sulfate). After hybridization, sections were washed 3 times for 5 minutes each in 4× saline-sodium citrate (SSC). Subsequently, sections were washed sequentially in 2× SSC containing 0.3% Tween-20 and 0.2× SSC containing 0.3% Tween-20 3 times each for 15 minutes each at 68°C. Sections were washed in 0.1× SSC containing 0.3% Tween-20 for 15 minutes followed by 3 washes of 5 minutes each in 0.1M PBS containing 0.3% Tween-20 at RT. The sections were then incubated with blocking solution (1× PBS, 2 mg/mL BSA, 0.3% Tween-20, and 10% normal sheep serum) for 1 hour at RT, followed by incubation with alkaline phosphatase-conjugated sheep anti-DIG Fab fragments (1:1,000 diluted in blocking solution, Roche 11093274910) overnight at 4°C. Hybridization signals were detected by incubating the sections in nitro blue tetrazolium and 5-bromo-4-chloro-3-indolyl phosphate (NBT/BCIP, 34042, Thermo Scientific) for 2 hours at RT and then counterstained with Nuclear Fast Red (H3403, Vector Laboratories; Burlingame, CA) for 5 minutes at RT. The differential interference contrast (DIC) images displayed in the main text were acquired with a C2 Nikon microscope equipped with a 20× and 60× oil objective. Sections in S5 Fig were observed and photographed on Zeiss Axioskop2 mot plus microscope equipped with a 40× objective. Control experiments (sense probe) were conducted simultaneously with identical procedure and conditions.

### Sequence alignment

The sea lamprey TAAR348 sequence (348 amino acid residues) was aligned with the sea lamprey TAAR346a sequence (346 amino acid residues) using CLUSTAL X 1.83 with default parameters. The transmembrane (TM) segments, extracellular loops (ECLs), and intracellular loops (ICLs) of TAAR348 and TAAR346a were predicted by TMpred program. The conserved residues of the aminergic ligand motif, TAAR fingerprint, and rhodopsin-type GPCRs were predicted based on previously described approaches [58, 59].

## Supporting information

S1 Fig Expanded view of semi-logarithmic plot of EOG amplitude (mean ± SEM) elicited by spermine (10^−15^ M to 10^−12^ M) in pre-ovulatory female (*n* = 10) and pre-spermiating male (*n* = 11) sea lampreys. (A) Expanded view of Fig 1C. The EOG response to spermine at each concentration was blank-subtracted and normalized to the response of 10^−5^ M _L_-arginine (standard) for each fish. (B) The EOG response to spermine at each concentration expressed as a percentage of the response to blank (vehicle in charcoal-filtered water handled in the same way as stimulus solution but without the addition of spermine) for each fish. The dashed line represents 100% of the blank. Deviation above the dashed line indicates detection of spermine different than the blank. Underlying data are available in S1 Data. EOG, electro-olfactogram.
(TIF)

S2 Fig Two-choice maze used to evaluate behavioral responses of sea lamprey to test stimuli. The 2 black circles represent test stimuli administration points. The large dashed lines represent flow boards used to reduce water turbulence. The small dashed lines represent fine mesh used to restrict the movement of the sea lamprey. The gray rectangle represents the release cage. Arrow represents the direction of water flow (0.07 m s^−1^ ± 0.01). Scale bar: 1 m.
(TIF)

S3 Fig Spermine induced higher luciferase activity in TAAR348 compared with sea lamprey ORs or V1Rs. HEK293T cells were incubated with OR, TAAR, or V1R plasmids or mock (empty vector) along with a CRE-luciferase reporter vector for 48 hours and subsequently stimulated with 10 or 100 μM spermine for 4 hours. Luciferase activity was indicated by the luminescence value and was normalized to the responses from the control stimuli DMSO (mean ± SEM, *n* = 2). Underlying data are available in S1 Data. CRE, cyclic-adenosine monophosphate response element; HEK293T, human embryonic kidney 293T; OR, odorant receptor; TAAR, trace amine-associated receptor; V1R, vomeronasal type 1 receptor.
(TIF)

S4 Fig Spermine induced higher luciferase activity than other tested amines in TAAR348. (A) HEK293T cells were incubated with TAAR348 plasmid or vehicle (empty vector) along with a CRE-luciferase reporter vector for 48 hours and subsequently stimulated with 10 μM of an amine for 4 hours. Luciferase activity was indicated by luminescence value and was normalized to the responses to the control stimuli DMSO (mean ± SEM, *n* = 2). (B) Spermine induced dose-dependent activity in HEK293T cells expressing TAAR348. HEK293T cells were incubated with TAAR348 plasmid or vehicle (empty vector) along with a CRE-luciferase reporter vector for 48 hours, stimulated with increasing concentrations of the indicated amine, and assayed for luciferase activity. Luciferase activity was indicated by the luminescence value and was normalized to the responses from the control stimuli DMSO (mean ± SEM). The sample size is indicated by the number in the parentheses. Underlying data are available in S1 Data. CRE, cyclic-adenosine monophosphate response element; HEK293T, human embryonic kidney 293T; TAAR, trace amine-associated receptor.
(TIF)

S5 Fig Representative olfactory receptor neurons with transcripts of sea lamprey *taar348*. *taar348* mRNA positive cells are labeled with a DIG-labeled antisense RNA probe in the cross-sections of the main olfactory epithelium of adult male and female sea lampreys. These cells are denoted with purple stain (NBT/BCIP) and black arrows. Sections were counterstained with Nuclear Fast Red. Black melanophores in the lamina propria are characteristic of sea lamprey olfactory epithelia. Images were acquired with a Zeiss Axioskop2 mot plus microscope equipped with a 40× Plan-Neuoflaur objective. Scale bar: 50 μm. LP, lamina propria; LU, lumen; NBT/BCIP, nitro blue tetrazolium and 5-bromo-4-chloro-3-indolyl phosphate; OE, olfactory epithelium.
(TIF)

S6 Fig Cyclen (SP20) identified as an antagonist of spermine receptor TAAR348 and nap-spermine (SP21) as another agonist. (A) To identify antagonists, HEK293T cells were reverse transfected with a TAAR348 plasmid and CRE-luciferase reporter vector, incubated for 48 hours, then stimulated with simultaneous application of 10-μM spermine along with 10 μM of a series spermine analogs (see list in S2 Table). After incubation for 4 hours, luciferase activity was assessed. Luciferase activity was normalized to the responses from the vehicle control stimulus DMSO (mean ± SEM, *n* = 2). Cyclen inhibited the spermine-induced luciferase activity in TAAR348-expressing HEK293T cells. (B) To assess agonist activity, HEK293T cells were reverse transfected with a TAAR348 plasmid and CRE-luciferase reporter vector and incubated for 48 hours. The cells were then stimulated with 10 μM of the spermine analogs alone (see list in S2 Table), incubated 4 hours, and then assayed for luciferase activity. Luciferase activity normalized to the responses from the control stimulus DMSO (mean ± SEM, *n* = 2). SP21 (Nap-spermine) was identified as a full agonist of TAAR348. Underlying data are available in S1 Data. CRE, cyclic-adenosine monophosphate response element; HEK293T, human embryonic kidney 293T; nap-spermine, 1-naphthylacetyl spermine; SP20, cyclen; TAAR, trace amine-associated receptor.
(TIF)

S7 Fig Sequence alignment of sea lamprey TAAR348 and TAAR346a. Sea lamprey TAAR348 (348 amino acid residues) was aligned with TAAR346a (346 amino acid residues) using CLUSTAL X 1.83 with default parameters. The amino acid sequences of the 2 receptors share 47% identity and 66% conservative substitutions. Marks for highly conserved amino acid substitutions: “*” residues which have a single, fully conserved residue; “:” residues with one of the following “strong” groups fully conserved: STA, NEQK, NHQK, NDEQ, QHRK, MILV, MILF, HY, FYW; and “.” residues with one of the following “weak” groups fully conserved: CSA, ATV, SAG, STNK, STPA, SGND, SNDEQK, NDEQHK, NEQHRK, FVLIM, HFY. We used positive scores from the Gonnet Pam250 matrix to define strong (score > 0.5) and weak groups (score ≤ 0.5). Color assignment was based on the amino acid residue profile specified in Clustal X. Pentagrams indicate broadly conserved residues in rhodopsin-type GPCRs; triangles indicate the aminergic ligand motif; squares indicate the characteristic fingerprint of TAARs. ATV, Alanine Threonine Valine; CSA, Cysteine Serine Alanine; ECL, extracellular loop; FYW, Phenylalanine Tyrosine Tryptophan; FVLIM, Phenylalanine Valine Leucine Isoleucine Methionine; GPCR, G-protein-coupled receptor; HFY, Histidine Phenylalanine Tyrosine; HY, Histidine Tyrosine; ICL, intracellular loop; MILF, Methionine Isoleucine Leucine Phenylalanine; MILV, Methionine Isoleucine Leucine Valine; NDEQ, Asparagine Aspartic acid Glutamic acid Glutamine; NDEQHK, Asparagine Aspartic acid Glutamic acid Glutamine Histidine Lysine; NEQHRK, Asparagine Glutamic acid Glutamine Histidine Arginine Lysine; NEQK, Asparagine Glutamic acid Glutamine Lysine; NHQK, Asparagine Histidine Glutamine Lysine; QHRK, Glutamine Histidine Arginine Lysine; SAG, Serine Alanine Glycine; SGND, Serine Glycine Asparagine Aspartic acid; SNDEQK, Serine Asparagine Aspartic acid Glutamic acid Glutamine Lysine; STA, Serine Threonine Alanine; STNK, Serine Threonine Asparagine Lysine; STPA, Serine Threonine Proline Alanine; TAAR, trace amine-associated receptor; TM, transmembrane region.
(TIF)

S1 Table The concentration of spermine in water conditioned with spermiating male and ovulatory female sea lampreys determined with UHPLC–MS/MS. The limit of spermine quantification with the UHPLC−MS/MS was 1.0 ng mL^−1^. The water samples were concentrated 50 times prior to subjecting them to the UHPLC−MS/MS, so the limit of spermine quantification of the water samples was 0.02 ng mL^−1^. ^†^The handling of sea lampreys most likely resulted in the incidental release of expressible milt into the water sample. Adult lamprey skin is slippery. To transfer lampreys in and out of water sampling buckets, one needs to grip both head and tail regions of the lamprey, and accidental pressure on the abdomen is difficult to avoid. N.D., not detectable; UHPLC−MS/MS, ultrahigh performance liquid chromatography-tandem mass spectrometry.
(DOCX)

S2 Table Spermine structural analogs screened as TAAR348 spermine receptor antagonists and agonists. CAS, Chemical abstract service; TAAR, trace amine-associated receptor.
(DOCX)

S1 Data Excel spreadsheet containing, in separate sheets, the underlying numerical data for figures in the main text and supplementary information. (XLSX)

## Abbreviations

BSA: bovine serum albumin
cAMP: cyclic-adenosine monophosphate
CRE: cyclic-adenosine monophosphate response element
DIC: differential interference contrast
DIG: digoxigenin
DMEM: Dulbecco’s Modified Eagle Medium
ECL: extracellular loop
EC_50_: half-maximal effective concentration
EGFP: enhanced green fluorescent protein
EOG: electro-olfactogram
ESI–MS/MS: electrospray ionization mass spectrometry
FBS: fetal bovine serum
GFP: green fluorescent protein
GPCR: G-protein-coupled receptor
HEK293T: human embryonic kidney 293T
HTS: high-throughput screening
ICL: intracellular loop
IC_50_: half-maximal inhibitory concentration
IS: internal standard
MRM: Multiple Reaction Monitoring
nap-spermine: 1-naphthylacetyl spermine
MS222: 3-aminobenzoic acid ethyl ester
NBT/BCIP: nitro blue tetrazolium and 5-bromo-4-chloro-3-indolyl phosphate
OR: odorant receptor
OSN: olfactory sensory neuron
RT: room temperature
Sp-*d*_8_: [^2^H_8_] -spermine
SSC: saline-sodium citrate
SV40: simian virus 40
TAAR: trace amine-associated receptor
TCA: trichloroacetic acid
TM: transmembrane
TR-FRET: time-resolved fluorescence energy transfer
UHPLC–MS/MS: ultrahigh performance liquid chromatography-tandem mass spectrometry
V1R: vomeronasal type 1 receptor
3kPZS: 3-keto petromyzonol sulfate
