# Supplementary figures and images for: Spermine in semen of male sea lamprey acts as a sex pheromone
Source: PLoS Biol. 2019 Jul 9;17(7):e3000332. doi: 10.1371/journal.pbio.3000332 (PMC6615597; doi:10.1371/journal.pbio.3000332)

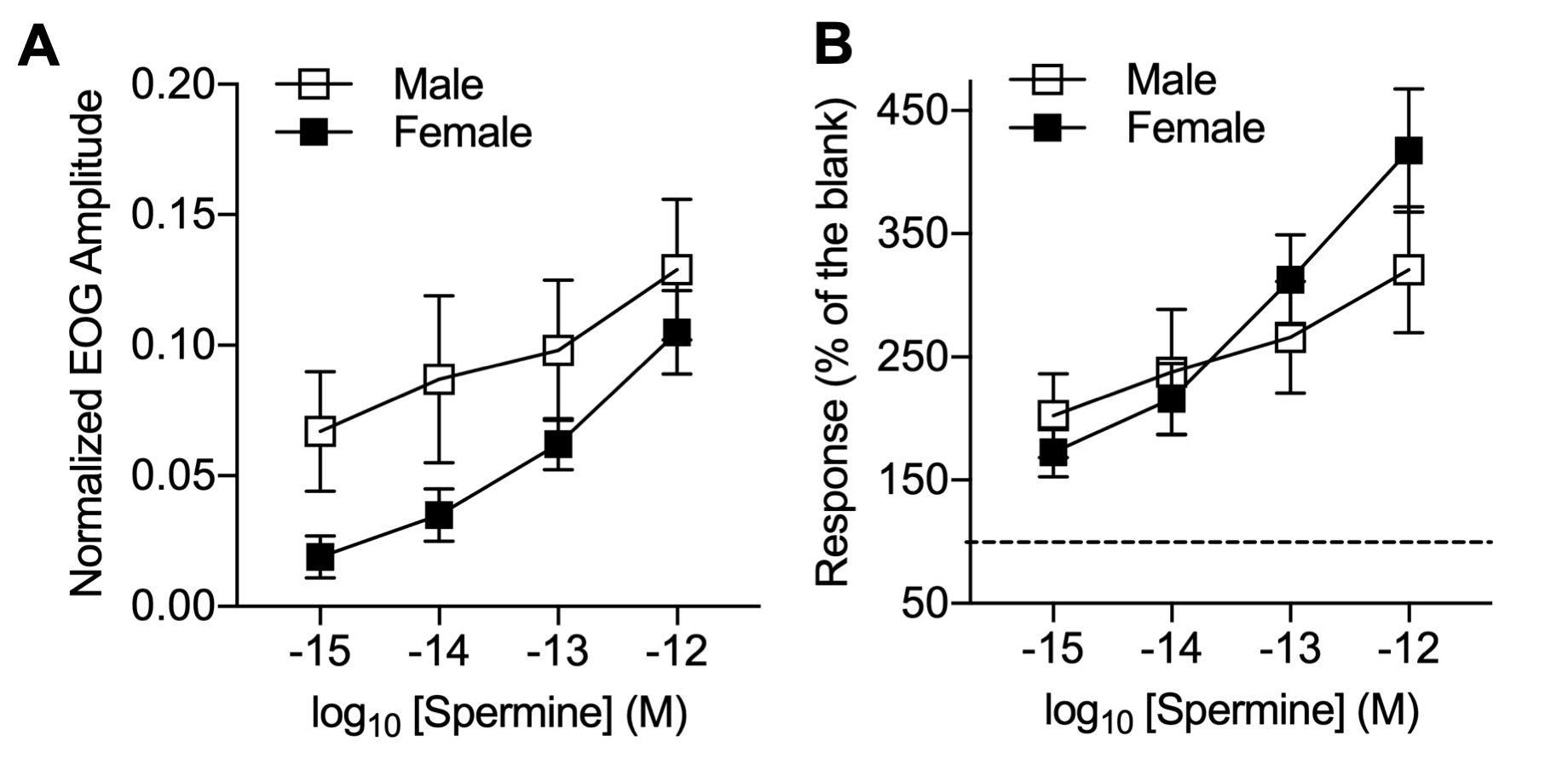

Supplement: S1 Fig — (A) Expanded view of Fig 1C. The EOG response to spermine at each concentration was blank-subtracted and normalized to the response of 10−5 M L-arginine (standard) for each fish. (B) The EOG response to spermine at each concentration expressed as a percentage of the response to blank (vehicle in charcoal-filtered water handled in the same way as stimulus solution but without the addition of spermine) for each fish. The dashed line represents 100% of the blank. Deviation above the dashed line indicates detection of spermine different than the blank. Underlying data are available in S1 Data. EOG, electro-olfactogram. (TIF) [file pbio.3000332.s001.tif]

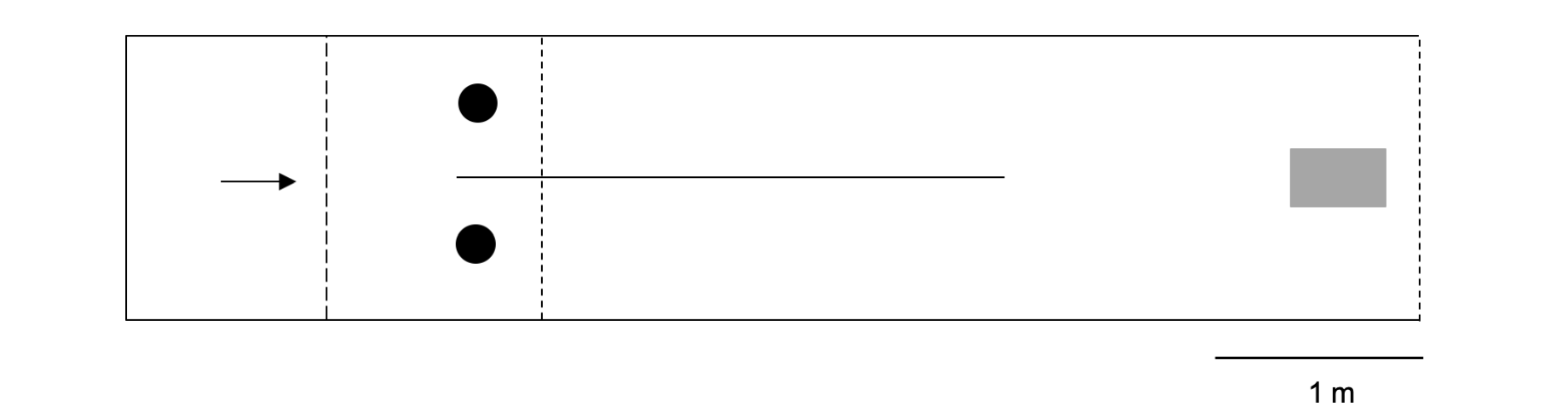

Supplement: S2 Fig — The 2 black circles represent test stimuli administration points. The large dashed lines represent flow boards used to reduce water turbulence. The small dashed lines represent fine mesh used to restrict the movement of the sea lamprey. The gray rectangle represents the release cage. Arrow represents the direction of water flow (0.07 m s−1 ± 0.01). Scale bar: 1 m. (TIF) [file pbio.3000332.s002.tif]

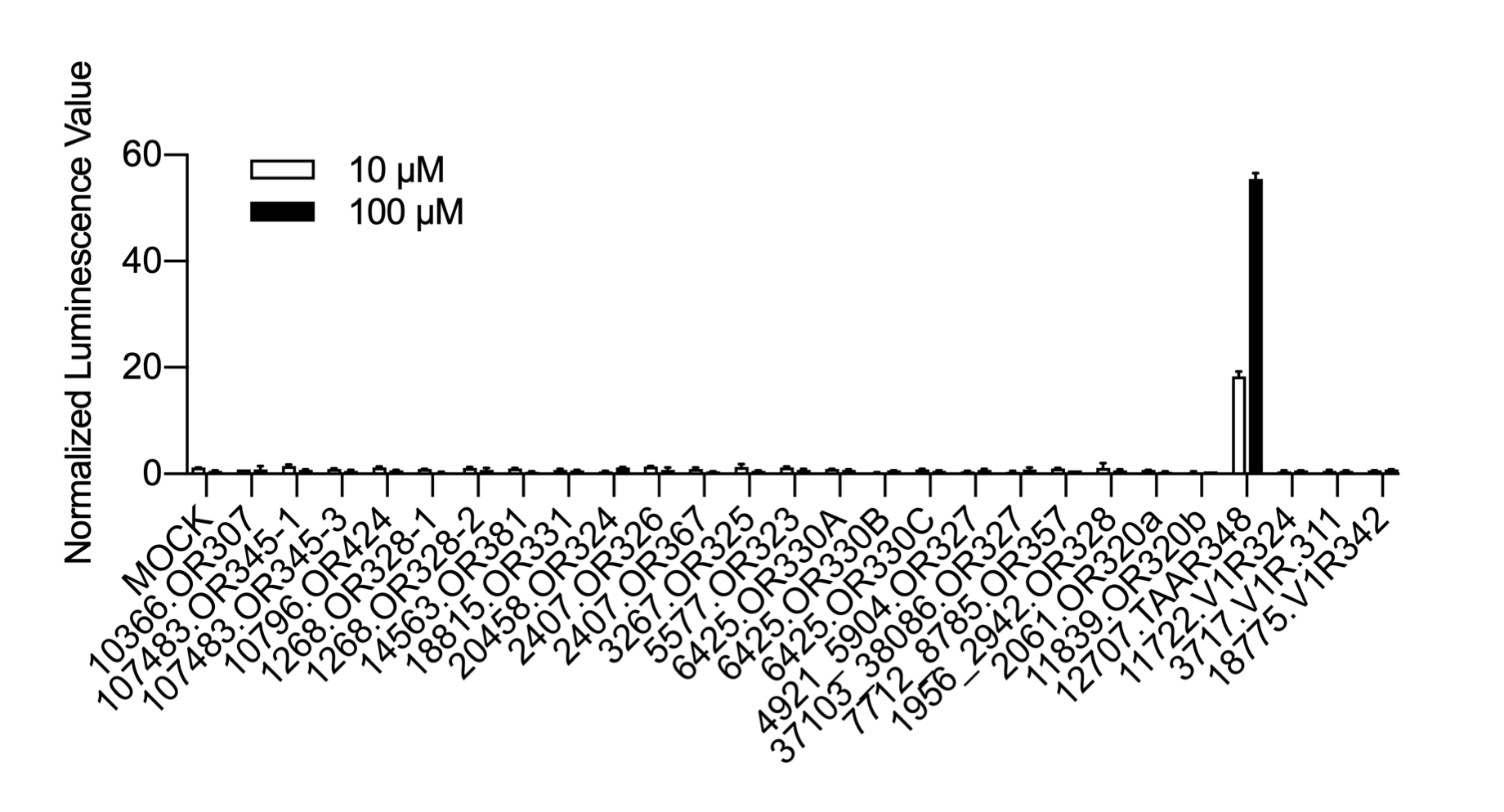

Supplement: S3 Fig — HEK293T cells were incubated with OR, TAAR, or V1R plasmids or mock (empty vector) along with a CRE-luciferase reporter vector for 48 hours and subsequently stimulated with 10 or 100 μM spermine for 4 hours. Luciferase activity was indicated by the luminescence value and was normalized to the responses from the control stimuli DMSO (mean ± SEM, n = 2). Underlying data are available in S1 Data. CRE, cyclic-adenosine monophosphate response element; HEK293T, human embryonic kidney 293T; OR, odorant receptor; TAAR, trace amine-associated receptor; V1R, vomeronasal type 1 receptor. (TIF) [file pbio.3000332.s003.tif]

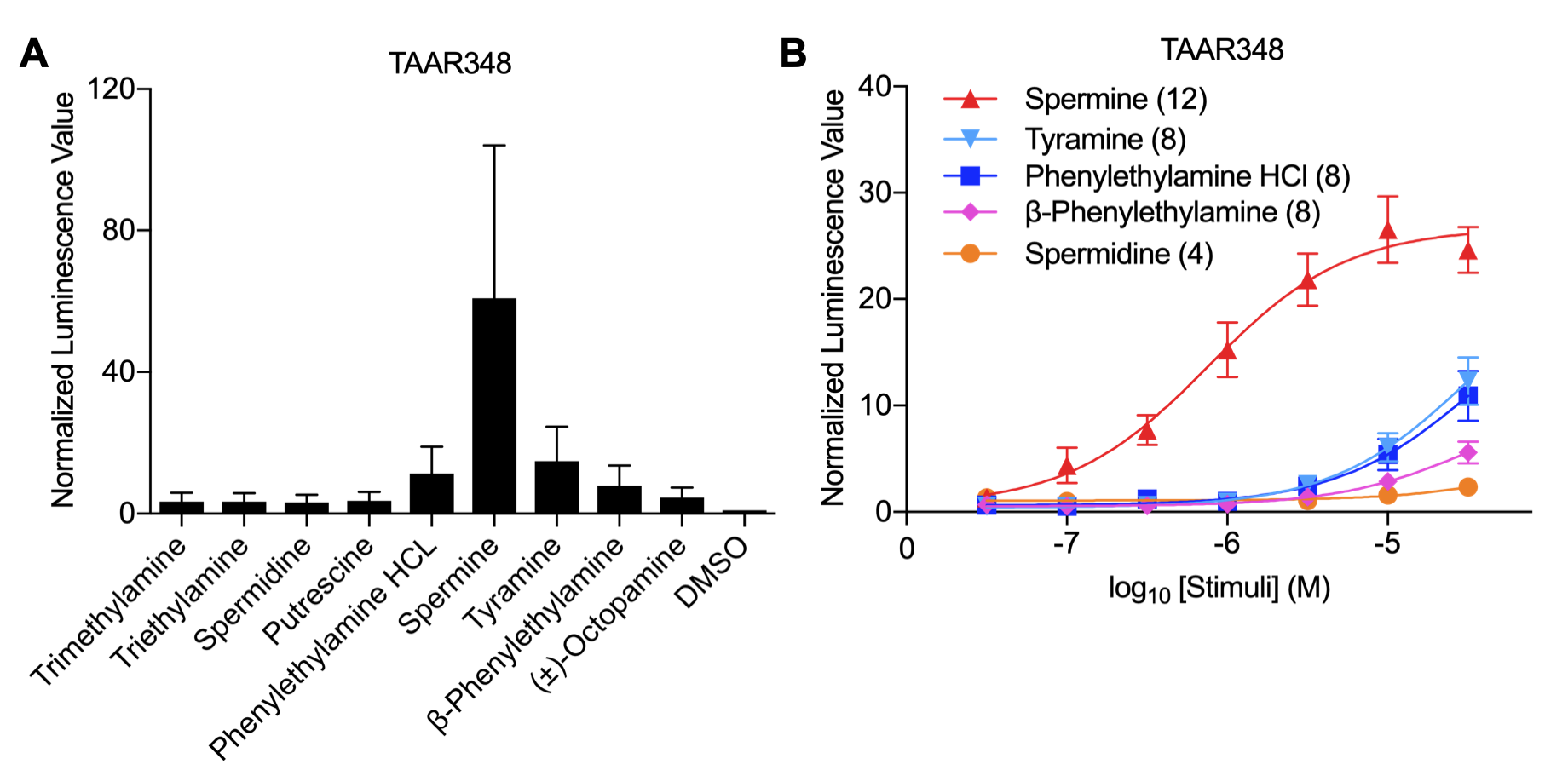

Supplement: S4 Fig — (A) HEK293T cells were incubated with TAAR348 plasmid or vehicle (empty vector) along with a CRE-luciferase reporter vector for 48 hours and subsequently stimulated with 10 μM of an amine for 4 hours. Luciferase activity was indicated by luminescence value and was normalized to the responses to the control stimuli DMSO (mean ± SEM, n = 2). (B) Spermine induced dose-dependent activity in HEK293T cells expressing TAAR348. HEK293T cells were incubated with TAAR348 plasmid or vehicle (empty vector) along with a CRE-luciferase reporter vector for 48 hours, stimulated with increasing concentrations of the indicated amine, and assayed for luciferase activity. Luciferase activity was indicated by the luminescence value and was normalized to the responses from the control stimuli DMSO (mean ± SEM). The sample size is indicated by the number in the parentheses. Underlying data are available in S1 Data. CRE, cyclic-adenosine monophosphate response element; HEK293T, human embryonic kidney 293T; TAAR, trace amine-associated receptor. (TIF) [file pbio.3000332.s004.tif]

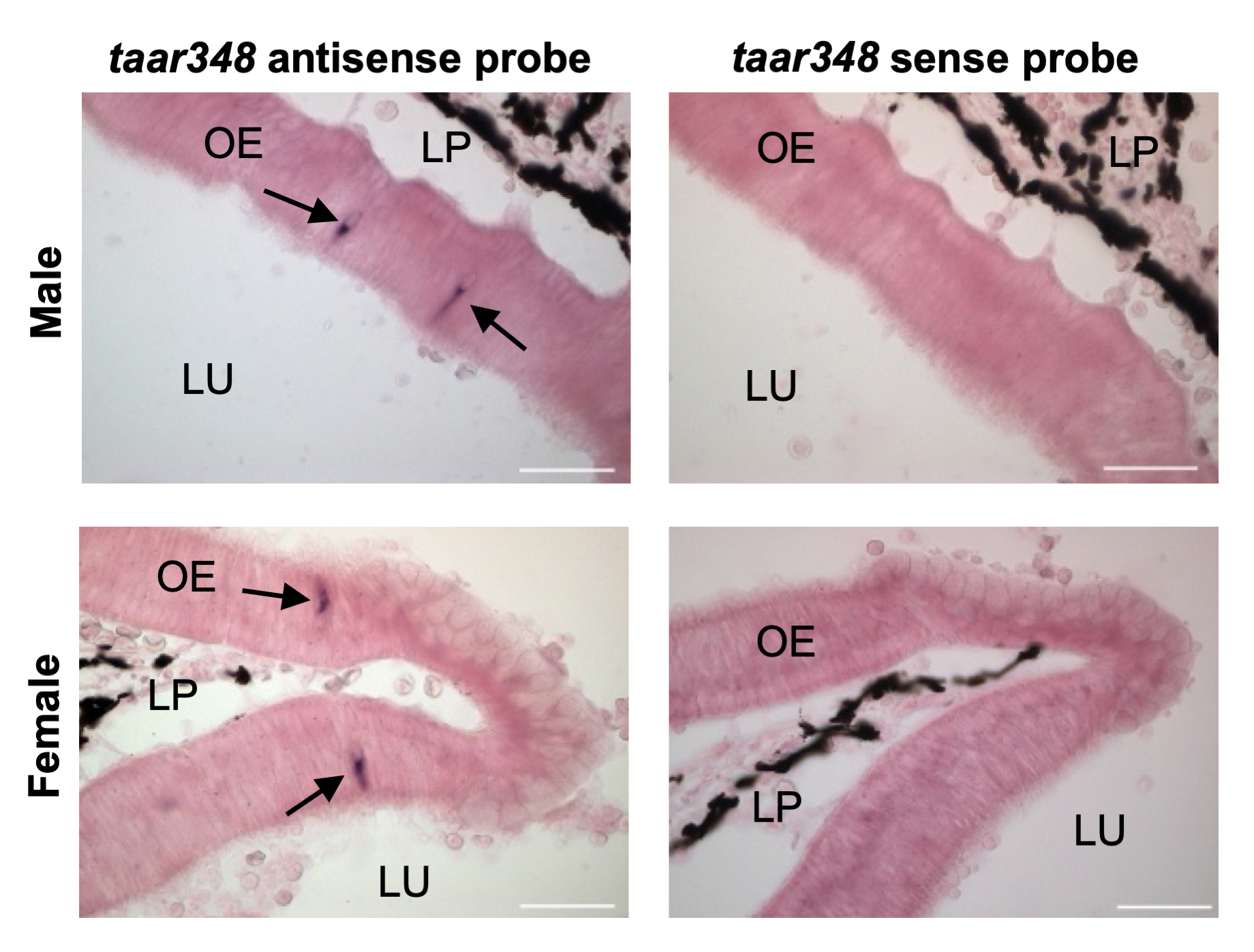

Supplement: S5 Fig — taar348 mRNA positive cells are labeled with a DIG-labeled antisense RNA probe in the cross-sections of the main olfactory epithelium of adult male and female sea lampreys. These cells are denoted with purple stain (NBT/BCIP) and black arrows. Sections were counterstained with Nuclear Fast Red. Black melanophores in the lamina propria are characteristic of sea lamprey olfactory epithelia. Images were acquired with a Zeiss Axioskop2 mot plus microscope equipped with a 40× Plan-Neuoflaur objective. Scale bar: 50 μm. LP, lamina propria; LU, lumen; NBT/BCIP, nitro blue tetrazolium and 5-bromo-4-chloro-3-indolyl phosphate; OE, olfactory epithelium. (TIF) [file pbio.3000332.s005.tif]

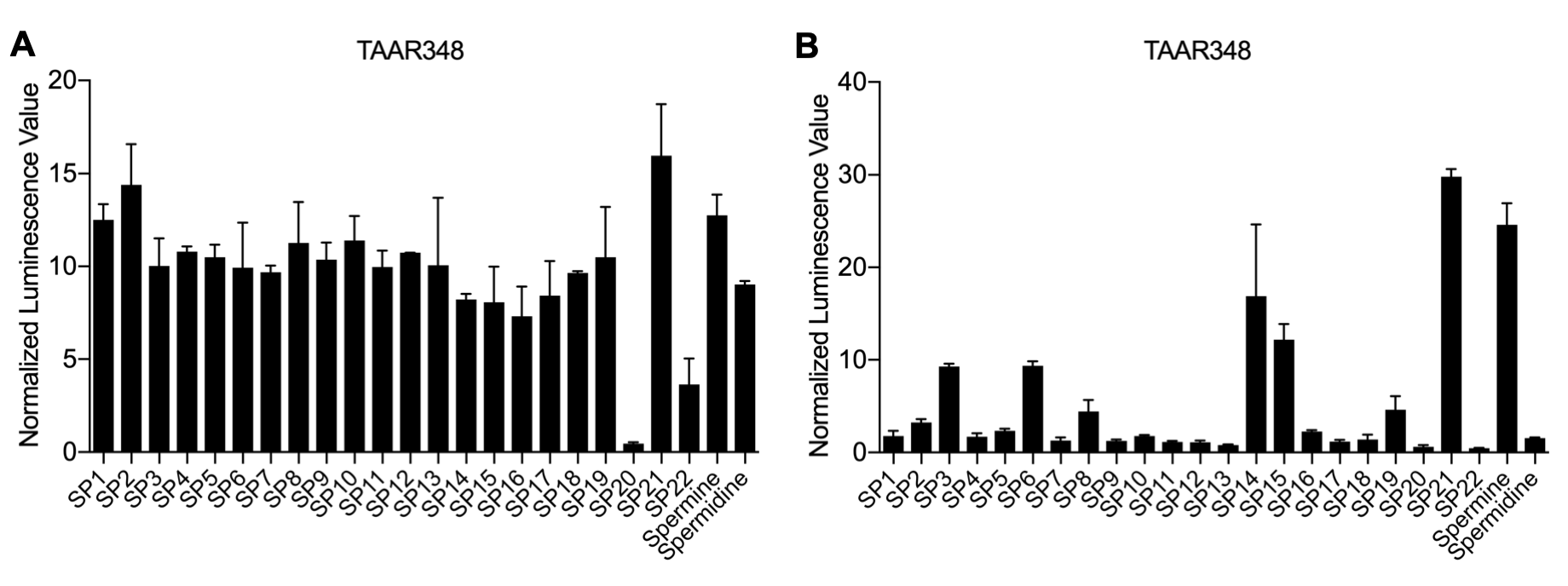

Supplement: S6 Fig — (A) To identify antagonists, HEK293T cells were reverse transfected with a TAAR348 plasmid and CRE-luciferase reporter vector, incubated for 48 hours, then stimulated with simultaneous application of 10-μM spermine along with 10 μM of a series spermine analogs (see list in S2 Table). After incubation for 4 hours, luciferase activity was assessed. Luciferase activity was normalized to the responses from the vehicle control stimulus DMSO (mean ± SEM, n = 2). Cyclen inhibited the spermine-induced luciferase activity in TAAR348-expressing HEK293T cells. (B) To assess agonist activity, HEK293T cells were reverse transfected with a TAAR348 plasmid and CRE-luciferase reporter vector and incubated for 48 hours. The cells were then stimulated with 10 μM of the spermine analogs alone (see list in S2 Table), incubated 4 hours, and then assayed for luciferase activity. Luciferase activity normalized to the responses from the control stimulus DMSO (mean ± SEM, n = 2). SP21 (Nap-spermine) was identified as a full agonist of TAAR348. Underlying data are available in S1 Data. CRE, cyclic-adenosine monophosphate response element; HEK293T, human embryonic kidney 293T; nap-spermine, 1-naphthylacetyl spermine; SP20, cyclen; TAAR, trace amine-associated receptor. (TIF) [file pbio.3000332.s006.tif]

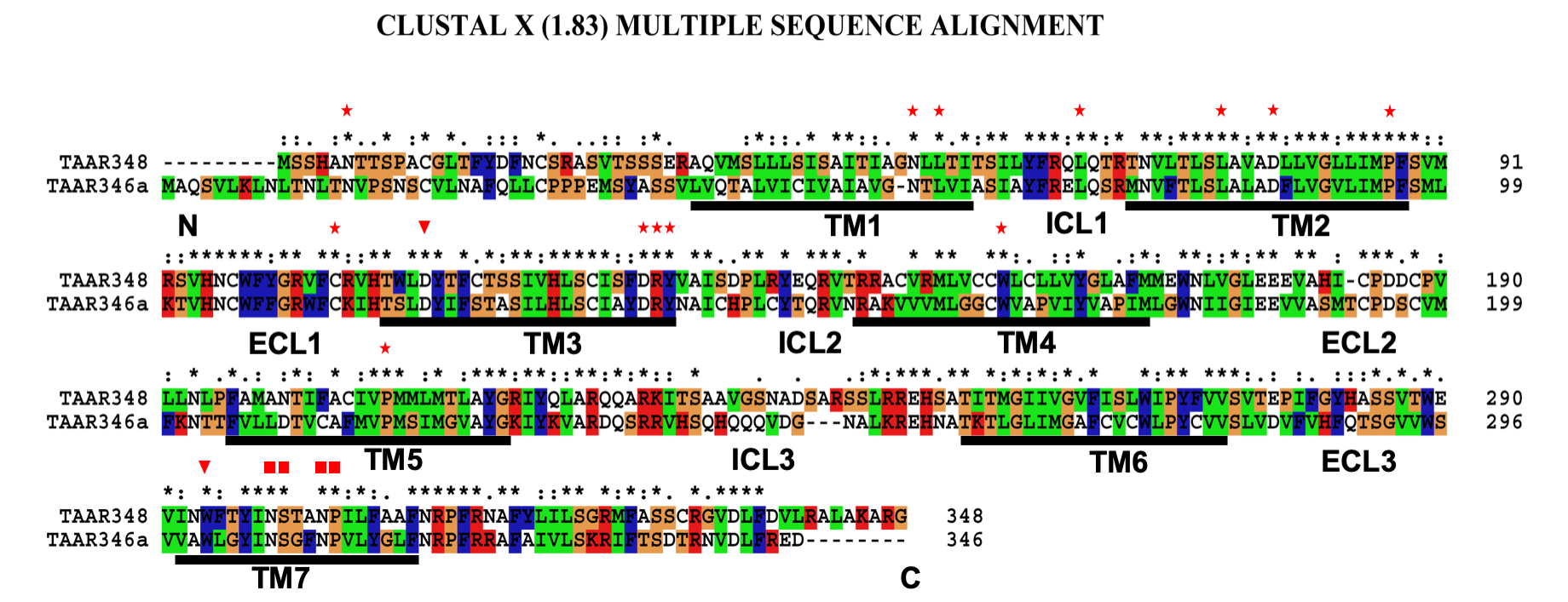

Supplement: S7 Fig — Sea lamprey TAAR348 (348 amino acid residues) was aligned with TAAR346a (346 amino acid residues) using CLUSTAL X 1.83 with default parameters. The amino acid sequences of the 2 receptors share 47% identity and 66% conservative substitutions. Marks for highly conserved amino acid substitutions: “*” residues which have a single, fully conserved residue; “:” residues with one of the following “strong” groups fully conserved: STA, NEQK, NHQK, NDEQ, QHRK, MILV, MILF, HY, FYW; and “.” residues with one of the following “weak” groups fully conserved: CSA, ATV, SAG, STNK, STPA, SGND, SNDEQK, NDEQHK, NEQHRK, FVLIM, HFY. We used positive scores from the Gonnet Pam250 matrix to define strong (score > 0.5) and weak groups (score ≤ 0.5). Color assignment was based on the amino acid residue profile specified in Clustal X. Pentagrams indicate broadly conserved residues in rhodopsin-type GPCRs; triangles indicate the aminergic ligand motif; squares indicate the characteristic fingerprint of TAARs. ATV, Alanine Threonine Valine; CSA, Cysteine Serine Alanine; ECL, extracellular loop; FYW, Phenylalanine Tyrosine Tryptophan; FVLIM, Phenylalanine Valine Leucine Isoleucine Methionine; GPCR, G-protein-coupled receptor; HFY, Histidine Phenylalanine Tyrosine; HY, Histidine Tyrosine; ICL, intracellular loop; MILF, Methionine Isoleucine Leucine Phenylalanine; MILV, Methionine Isoleucine Leucine Valine; NDEQ, Asparagine Aspartic acid Glutamic acid Glutamine; NDEQHK, Asparagine Aspartic acid Glutamic acid Glutamine Histidine Lysine; NEQHRK, Asparagine Glutamic acid Glutamine Histidine Arginine Lysine; NEQK, Asparagine Glutamic acid Glutamine Lysine; NHQK, Asparagine Histidine Glutamine Lysine; QHRK, Glutamine Histidine Arginine Lysine; SAG, Serine Alanine Glycine; SGND, Serine Glycine Asparagine Aspartic acid; SNDEQK, Serine Asparagine Aspartic acid Glutamic acid Glutamine Lysine; STA, Serine Threonine Alanine; STNK, Serine Threonine Asparagine Lysine; STPA, Serine Threonine Proline Alanine; T [file pbio.3000332.s007.tif]
