# Supplementary material for: Spermine in semen of male sea lamprey acts as a sex pheromone
Source: PLoS Biol. 2019 Jul 9;17(7):e3000332. doi: 10.1371/journal.pbio.3000332 (PMC6615597; doi:10.1371/journal.pbio.3000332)
Supplement: S1 Table — The limit of spermine quantification with the UHPLC−MS/MS was 1.0 ng mL−1. The water samples were concentrated 50 times prior to subjecting them to the UHPLC−MS/MS, so the limit of spermine quantification of the water samples was 0.02 ng mL−1. †The handling of sea lampreys most likely resulted in the incidental release of expressible milt into the water sample. Adult lamprey skin is slippery. To transfer lampreys in and out of water sampling buckets, one needs to grip both head and tail regions of the lamprey, and accidental pressure on the abdomen is difficult to avoid. N.D., not detectable; UHPLC−MS/MS, ultrahigh performance liquid chromatography-tandem mass spectrometry. (DOCX) [file pbio.3000332.s008.docx]

| Sample ID | Concentration (ng mL^−1^) |
| --- | --- |
| Spermiating Male-1 | N.D. |
| Spermiating Male-2 | N.D. |
| Spermiating Male-3 | 0.12^†^ |
| Spermiating Male-4 | N.D. |
| Spermiating Male-5 | N.D. |
| Spermiating Male-6 | N.D. |
| Spermiating Male-7 | N.D. |
| Spermiating Male-8 | N.D. |
| Spermiating Male-9 | N.D. |
| Ovulatory Female-1 | N.D. |
| Ovulatory Female-2 | N.D. |
| Ovulatory Female-3 | N.D. |
| Ovulatory Female-4 | N.D. |
| Ovulatory Female-5 | N.D. |
| Ovulatory Female-6 | N.D. |
| Ovulatory Female-7 | N.D. |
| Ovulatory Female-8 | N.D. |
| Ovulatory Female-9 | N.D. |
| Ovulatory Female-10 | N.D. |
