# Supplementary material for: Spermine in semen of male sea lamprey acts as a sex pheromone
Source: PLoS Biol. 2019 Jul 9;17(7):e3000332. doi: 10.1371/journal.pbio.3000332 (PMC6615597; doi:10.1371/journal.pbio.3000332)
Supplement: S2 Table — CAS, Chemical abstract service; TAAR, trace amine-associated receptor. (DOCX) [file pbio.3000332.s009.docx]

| Analog ID | CAS* number | Common name |
| --- | --- | --- |
| SP1 | 109-76-2 | 1,3-Diaminopropane |
| SP2 | 110-60-1 | 1,4-Diaminobutane |
| SP3 | 462-94-2 | 1,5-Diaminopentane |
| SP4 | 646-19-5 | 1,7-Diaminoheptane |
| SP5 | 373-44-4 | 1,8-Diaminooctane |
| SP6 | 646-25-3 | 1,10-Diaminodecane |
| SP7 | 124-09-4 | Hexamethylenediamine |
| SP8 | 4605-14-5 | N,N′-Bis(3-aminopropyl)-1,3-propanediamine |
| SP9 | 4741-99-5 | N,N′-Bis(2-aminoethyl)-1,3-propanediamine |
| SP10 | 10563-26-5 | 1,2-Bis(3-aminopropylamino)ethane |
| SP11 | 7209-38-3 | 1,4-Bis(3-aminopropyl)piperazine |
| SP12 | 295-37-4 | 1,4,8,11-Tetraazacyclotetradecane |
| SP13 | 15439-16-4 | 1,4,8,12-Tetraazacyclopentadecane |
| SP14 | 113812-15-0 | N1,N12-Diethylspermine tetrahydrochloride |
| SP15 | 77928-70-2 | N1-Acetylspermine trihydrochloride |
| SP16 | 177213-61-5 | N4,N9-di-Boc-spermine |
| SP17 | 112-24-3 | Triethylenetetramine |
| SP18 | 112-57-2 | Tetraethylenepentamine |
| SP19 | 1310544-60-5 | N,N'-Bis(2-pyridylmethyl)-1,2-ethylenediamine tetrahydrochloride |
| SP20 | 294-90-6 | Cyclen (1,4,7,10-Tetraazacyclododecane) |
| SP21 | 122306-11-0 | Nap-spermine (1-Naphthylacetyl spermine) |
| SP22 | 295-14-7 | 1,4,7,10-Tetraazacyclotridecane |
